# Improved Dual Base Editor Systems (iACBEs) for Simultaneous Conversion of Adenine and Cytosine in the Bacterium Escherichia coli

**DOI:** 10.1128/mbio.02296-22

**Published:** 2023-01-10

**Authors:** Rahul Mahadev Shelake, Dibyajyoti Pramanik, Jae-Yean Kim

**Affiliations:** a Division of Applied Life Science (BK21 Four Program), Plant Molecular Biology and Biotechnology Research Center, Gyeongsang National University, Jinju, South Korea; b Division of Life Science, Gyeongsang National University, Jinju, South Korea; c Nulla Bio Inc., Jinju, South Korea; The Rockefeller University; Weill Cornell Medicine

**Keywords:** CRISPR, base editing, dual base editor, genome editing, microbial engineering

## Abstract

Genome-editing (GE) techniques like base editing are ideal for introducing novel gain-of-function mutations and *in situ* protein evolution. Features of base editors (BEs) such as higher efficacy, relaxed protospacer adjacent motif (PAM), and a broader editing window enables diversification of user-defined targeted locus. Cytosine (CBE) or adenine (ABE) BEs alone can only alter C-to-T or A-to-G in target sites. In contrast, dual BEs (ACBEs) can concurrently generate C-to-T and A-to-G modifications. Although BE tools have recently been applied in microbes, there is no report of ACBE for microbial GE. In this study, we engineered four improved ACBEs (iACBEs) tethering highly active CBE and ABE variants that can introduce synchronized C-to-T and A-to-G mutations in targeted loci. iACBE4 generated by evoCDA1-ABE9e fusion demonstrated a broader editing window (positions −6 to 15) and is also compatible with the multiplex editing approach in Escherichia coli. We further show that the iACBE4-NG containing PAM-relaxed nCas9-NG expands the targeting scope beyond NGG (N-A/G/C/T) PAM. As a proof-of-concept, iACBE was effectively utilized to identify previously unknown mutations in the *rpoB* gene, conferring gain-of-function, i.e., rifampicin resistance. The iACBE tool would expand the CRISPR-GE toolkit for microbial genome engineering and synthetic biology.

## INTRODUCTION

Several genetic engineering tools based on CRISPR/Cas system have recently been developed and adopted to edit genetic information in different organisms (1–3). Base editors (BEs) are one of the emerging CRISPR technologies that offer unique features compared to traditional CRISPR/Cas9 uses. For example, the primary outcome of CRISPR/Cas9 application includes the insertion or deletion that typically introduces frameshift mutations leading to the generation of a premature stop codon (knockout) or nonfunctional peptides. In contrast, BE tools generate base substitutions using deaminases tethered with Cas9 variants and DNA repair components to create point mutations, allowing *in vivo* protein evolution (4). Moreover, double-strand breaks (DSBs)-mediated gene disruption by CRISPR/Cas9 is primarily the outcome of the error-prone nonhomologous end-joining (NHEJ) pathway. The active NHEJ pathway is absent in most bacteria, thus impeding the CRISPR/Cas9 use. Even when NHEJ is present in some bacteria, it is not as active as in mammalian cells. In this scenario, DSB-free BEs are promising tools for targeted base editing in bacteria.

Among the available BE arsenals, the main BE tools comprise cytosine base editor (CBE) (5, 6) and adenine base editor (ABE) (7) that enable C:G to T:A and A:T to G:C conversion, respectively. Individual CBE or ABE can generate mutations in only a single type of nucleotide base-pair. To broaden the range of DNA modifications, simultaneous C-to-T and A-to-G substitution was achieved by developing dual (adenine-cytosine) base editors (ACBEs) by combining the deaminases and related BE components of CBEs and ABEs (4, 8–14). ACBE-mediated simultaneous conversion of two types of DNA base-pairs on the same target site enables greater diversification of desired alleles in directed evolution studies. Saturated mutagenesis of target genes by ACBE is an efficient way for studying the genotype-phenotype relationships.

Although BE tools have recently been reported for microbial genome engineering (15), to the best of our knowledge, there is no report of ACBE tool establishment in microbial species, including bacteria. Also, most of the earlier ACBE types consisted of Streptococcus pyogenes Cas9 (SpCas9, hereafter Cas9) nickase (nCas9, D10A) that recognizes NGG (N- A/G/C/T) as a protospacer adjacent motif (PAM), offering a limited target range. One of the significant challenges for applying highly active BE-based tools in bacteria is the adverse effects of overexpressed BE components on cell growth and survival (15–17). In some cases, the use of inducible or relatively weak constitutive promoters, adding a protein degradation (LVA) tag, replacing nCas9 with catalytically dead Cas9 (dCas9, D10A+H840A), or a combination of all the above factors helped to reduce the cytotoxicity permitting the BE applications in different microbial species, including E. coli, *Bacillus* sp., Pseudomonas sp., *Agrobacterium* spp., Paenibacillus polymyxa, *Streptomyces* spp., etc. (15–20). Moreover, highly active BE variants such as evoCDA1, ABE8e, and ABE9e would pose further difficulty due to their broader editing window and faster base conversion rate than their predecessors (21–23). Recently, a platform named *in vivo* rapid investigation of BE components in E. coli (IRI-CCE) (24) was developed that comprises an optimized set of promoter-terminator combinations allowing an appropriate amount of BE expression with higher editing activities at desired target sites in plasmid DNA or genomic loci.

This study describes the establishment of improved ACBE (iACBE) systems for bacterial use by employing the IRI-CCE platform in E. coli. We engineered a series of iACBEs by fusing different CBE (PmCDA1, evoCDA1 [evolved version of PmCDA1], APOBEC3A) and ABE (ABE8e, ABE9e) variants to accomplish efficient A/C base editing in plasmid and chromosomal targets in E. coli. Furthermore, we comprehensively analyzed the applicability of iACBE4, the most active iACBE type comprising evoCDA1-ABE9e, by targeting 14 genomic target sites. To broaden the targeting range of iACBE4, nCas9-NG, a PAM-flexible Cas9 variant recognizing NGN as a PAM (25), was fused to generate an iACBE4-NG tool that enabled the editing of targets with non-NGG PAM. To avoid self-targeting frequency while using nCas9-NG, the modified single guide RNA (sgRNA) scaffold starting with “GCCCC” (esgRNA) (26) was evaluated. Using the optimized iACBE4 and iACBE4-NG, we targeted the RNA polymerase *rpoB* gene of E. coli implicated in rifampicin resistance (Rif^R^). The screening of generated *rpoB* mutants identified previously unknown mutations in single or multiple amino acids bestowing Rif^R^ in E. coli cells. This study demonstrates the iACBE potential for mutant library construction of desired genes enabling the quick evaluation of the correlation between genotype and gain-of-function.

## RESULTS

### Design and construction of improved dual base editor (iACBE) system.

**FIG 1:**
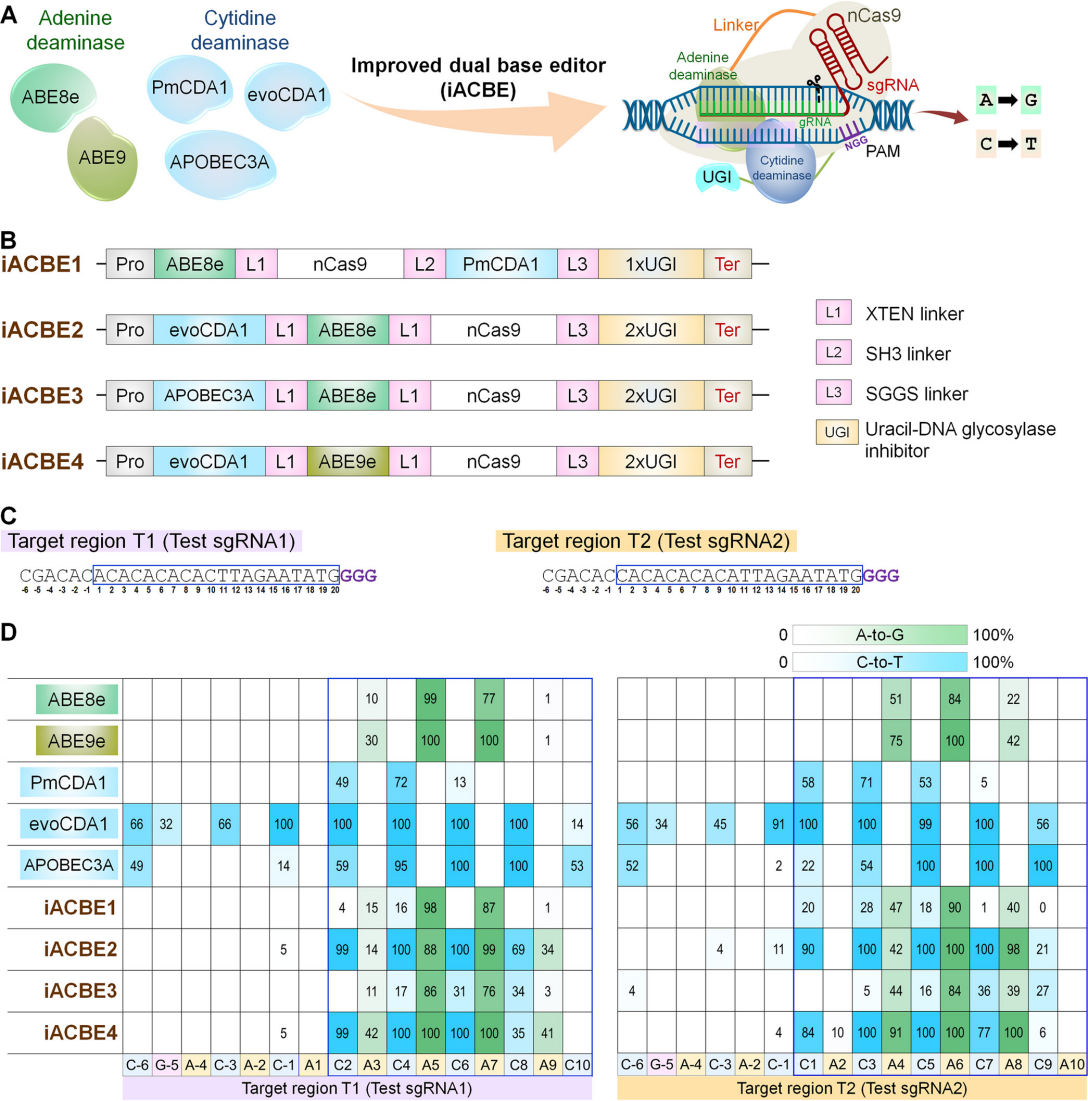
Improved dual base editor (iACBE) systems induce synchronized A-to-G and C-to-T mutations in E. coli cells. (A) Deaminases used for generation of improved dual base editors (iACBEs). (B) Schematic representation of the plasmid vectors iACBEs tested in the work for iACBE development. Pro, pGlpT promoter; Ter, L3S2P21 terminator; nCas9, SpCas9 nickase with D10A mutation. Different deaminases are explained in the main text. L1, XTEN liner; L2, SH3 linker; L3, SGGS linker. (C) Two independent sgRNAs, including alternate Cs at even (test sgRNA1) and odd (test gRNA2) positions spanning from 1 to 10 in the 20 bp gRNA-spacer were expressed using pAtU6 promoter. The numbers are assigned by counting the distal base as 1 from the protospacer adjacent motif (PAM), i.e., GGG counting as 21 to 23. (D) Base editing (C-to-T and A-to-G) activities by single and dual base editors (iACBEs). Heat map values show the mean percentage of base conversion at different positions in target sites calculated from four independent biological replicates. Nucleotide bases located withing the 20 bp gRNA highlighted inside the blue box. The base conversion rate was estimated using the online EditR tool.

To characterize a dual base editing system for the bacterial application, we designed four ACBE architectures combining ABE8e or ABE9e with either of the three different CBE versions, i.e., PmCDA1 and evoCDA1 and APOBEC3A (Fig. 1A). In the first set of mammalian and plant ACBE reports, editing efficiencies from A-to-G were lower than those of C-to-T, thereby reducing the occurrence of synchronized base editing (4, 8, 9, 11, 14). The heterodimer of adenosine deaminase (TadA-TadA*) opted for the ABE7.10 variant or ACBEs (7–14). The TadA-TadA* convert A-to-G within the editing window of positions 4 to 8 in the protospacer region (considering the PAM at positions 21 to 23) with relatively low efficiency (7). We reasoned that using highly active TadA in ACBE would boost the prospects of attaining concurrent base editing. Therefore, we chose monomeric ABE8e (23) and ABE9e (22) for bacterial ACBE designs, the most active TadA versions so far. The fusion of TadA to the N-terminus of nCas9 was chosen since C-terminal fusion was reported to lack activity in ACBEs (14).

Meanwhile, PmCDA1, a sea lamprey cytidine deaminase, mediates C-to-T conversion within 1 to 5 bases as a canonical editing window in the protospacer region (5). Similar to the earlier mammalian ACBEs (8, 9, 11), PmCDA1 with one copy of uracil-DNA glycosylase inhibitor (UGI) was fused to the C-terminus and ABE8e at the N-terminus of nCas9, creating iACBE version 1 (iACBE1) (Fig. 1B). The use of UGI increases the purity of C-to-T conversion at target sites, a strategy used in previous reports of CBEs and ACBEs. Next, we chose evoCDA1 or APOBEC3A as ABE8e partners by fusing both the deaminases to the N-terminus of Cas9, producing iACBE2 and iACBE3, respectively. Two UGI (2xUGI) copies were connected at the C-terminus of nCas9, likewise used in previously published reports. Both evoCDA1 and APOBEC3A exhibited broader editing windows in E. coli and other organisms (21, 24, 27). The iACBE4 construct was generated by combining the most active individual BE deaminases, i.e., evoCDA1 and ABE9e (Fig. 1B). An optimized combination of promoter-terminator (pGlpT-TerL3S2P21) that allowed nonlethal expression of single-function BEs (ABEs and CBEs) in IRI-CCE (24), was adopted for the expression of iACBEs with a single-plasmid system in E. coli.

### Establishment of iACBE system in E. coli.

To examine the synchronized base-editing activities of the four designed ACBE constructs, we performed editing tests at two sgRNA target regions (Target 1, T1 by Test sgRNA1; and Target 2, T2 by Test sgRNA2) (Fig. 1C). Individual constructs were transformed in E. coli, and plasmids isolated from four independent clones were investigated for mutagenesis in targeted regions by Sanger sequencing and the online EditR tool (28). The dual A/C base editing frequency of all the tested iACBE versions was found to be 100% showing concurrent A-to-G and C-to-T edits at one or more positions of As and Cs in the protospacer region (Fig. 1D).

**FIG 2:**
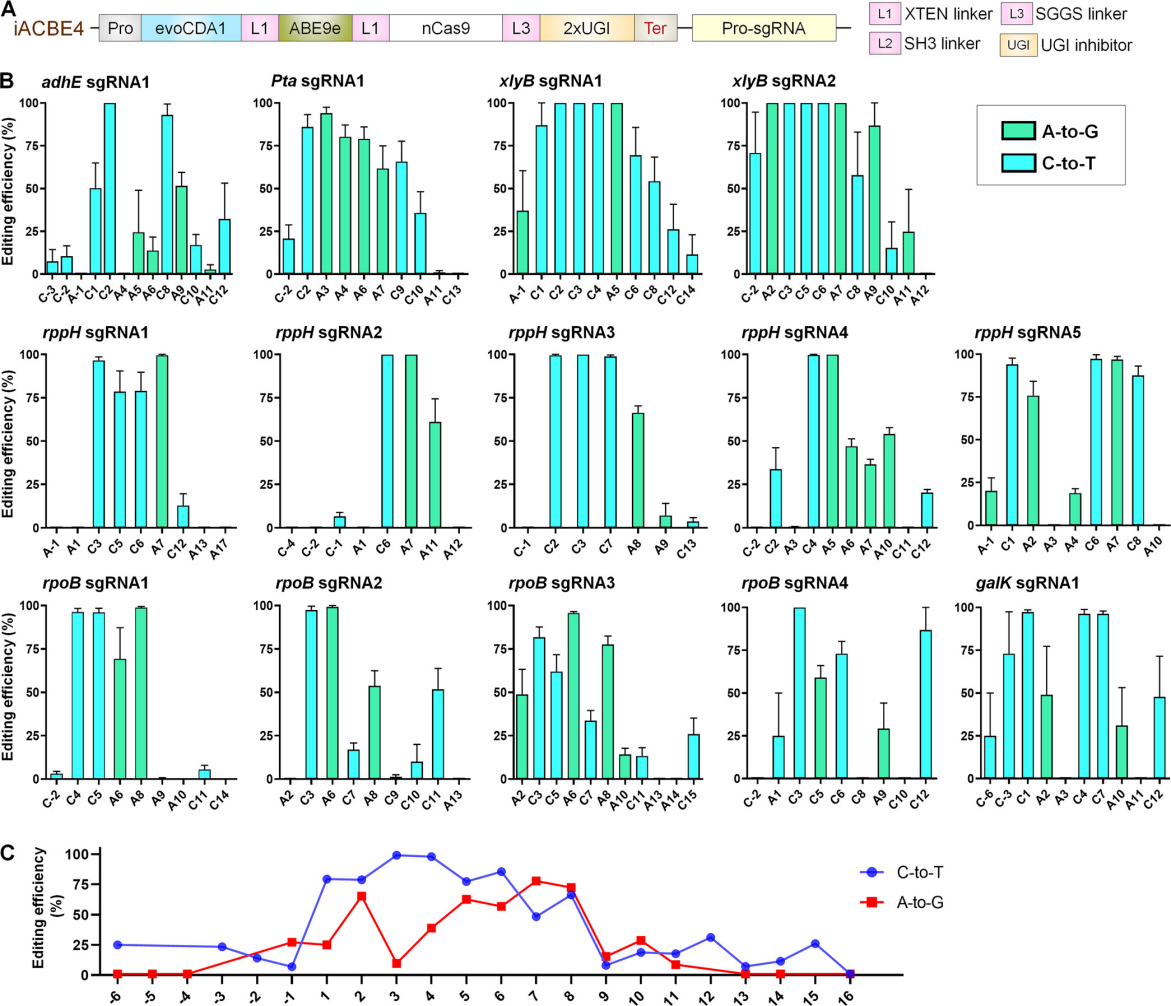
Determination of base editing efficiency of iACBE4 on target sites in the E. coli genome. (A) Architecture of plasmid constructs containing iACBE4 and sgRNA. iACBE4 cassette comprises of pGlpT promoter and L3S2P21 terminator. The sgRNAs were expressed using pAtU6 promoter. (B) Simultaneous C-to-T and A-to-G editing activities by iACBE4 at tested genomic target sites with different PAMs. The graph bar shows the mean of percentage values, and error bars indicate the standard error of the mean (mean ± SEM) of four independent biological replicates. The base conversion rate was estimated using the online tool EditR. (C) Merged data of average C-to-T and A-to-G efficiency from panel B. Mean values were plotted for each position separately.

Each ACBE type showed a variable range of editing efficiencies for specific A/C positions in the targeted region. Apparently, iACBE1, iACBE2, and iACBE4 commonly exhibited base-editing features similar to corresponding single-function BEs achieving 100% mutation at a minimum of one A/C position in the target region. While the on-target editing pattern of iACBE3 showed reduced concurrent A-to-G and C-to-T editing activities compared to their single BE counterparts (ABE8e or ABPOBE3A). Considering the combined data estimated in IRI-CCE platform of two tested targets with a minimum 5% base conversion rate, editing window length for ABE8e, ABE9e, PmCDA1, evoCDA1, and APOBEC3A spanned from 3 to 8, 3 to 8, 1 to 7, −6 to 10, −6 to 10, respectively (24). In the case of ACBEs, all four iACBE versions exhibited similar editing window lengths for A-to-G edits to that of ABEs, i.e., positions 3 to 8. C-to-T conversion window positioned from 1 to 7, -1 to 9, 3 to 9, and -1 to 9 for iACBE1 to 4, respectively (Fig. 1D). The lower amount of A/C base editing by iACBE3 was found in the editable window compared to ABE8e (range: 11% to 86% versus 10% to 99%) and APOBEC3A (0% to 36% versus 14% to 100%). Analysis of iACBE2 consisting of catalytically inactive dCas9 fusion also showed similar editing features compared to nCas9-based iACBE2 (Fig. S1). Overall, iACBE2 and iACBE4 showed a broader window and were the most effective iACBEs for installing concurrent A/C mutations among the tested versions. We chose iACBE4 (evoCDA1-ABE9e) for further characterization in subsequent experiments (Fig. 2A).

10.1128/mbio.02296-22.1 FIG S1 Comparative analysis of iACBE2(nCas9) and iACBE2(dCas9)-mediated editing outcome at synthetic target regions. Target 1 and Target 2 were analyzed for editing by iACBE2 (ABE8e-evoCDA1) designed with two different Cas9 forms, i.e., nCas9(D10A) and dCas9 (D10A+H840A). (A) Concurrent A/C base editing at Target 1 region by iACBE2(nCas9). (B) Concurrent A/C base editing at Target 1 region by iACBE2(dCas9). (C) Concurrent A/C base editing at Target 2 region by iACBE2(nCas9). (D) Concurrent A/C base editing at Target 2 region by iACBE2(dCas9). Download FIG S1, TIFF file, 1.0 MB. Copyright © 2023 Shelake et al. 2023 Shelake et al. https://creativecommons.org/licenses/by/4.0/ This content is distributed under the terms of the Creative Commons Attribution 4.0 International license.

### Evaluation of the editing efficiency by iACBE4 on E. coli genomic sites.

Next, we thoroughly assessed iACBE4 performance for mutating the endogenous target sites in E. coli. For targeting genomic loci, we chose 14 target sites having different A/C sequence contexts across the protospacer regions (Table S1). The pGlpT-driven iACBE4 effectively modified the available A/Cs in the target regions of all the 14 tested sites with variable efficiency (Fig. 2B). Notably, iACBE4 showed a broader editing window length of 21 nucleotides (positions −6 to 15) (Fig. 2C). Higher coediting of A/C was observed in assessed sites at positions 1 to 8. Among them, A-to-G and C-to-T conversions by iACBE4 were highest at positions 4 to 8 and 1 to 9, respectively, within the target region. The editing pattern also confirmed the 100% editing of at least one A or C in all the 14 targets, similarly observed for plasmid targets. Although editing efficacies of base editing systems may vary due to several factors like target site, sequence context, and experimental conditions (29), our results demonstrate the functionality of iACBE4 architecture for co-editing of A/C in the genomic context of E. coli.

10.1128/mbio.02296-22.5 TABLE S1 Genomic target DNA sites with different Protospacer adjacent motifs (PAMs) analyzed for base conversion by iACBE4 and iACBE4-NG in Fig. 2 and Fig. 5, respectively. Download Table S1, DOCX file, 0.04 MB. Copyright © 2023 Shelake et al. 2023 Shelake et al. https://creativecommons.org/licenses/by/4.0/ This content is distributed under the terms of the Creative Commons Attribution 4.0 International license.

### iACBE4-mediated multiplex editing allows multisite editing in E. coli.

**FIG 3:**
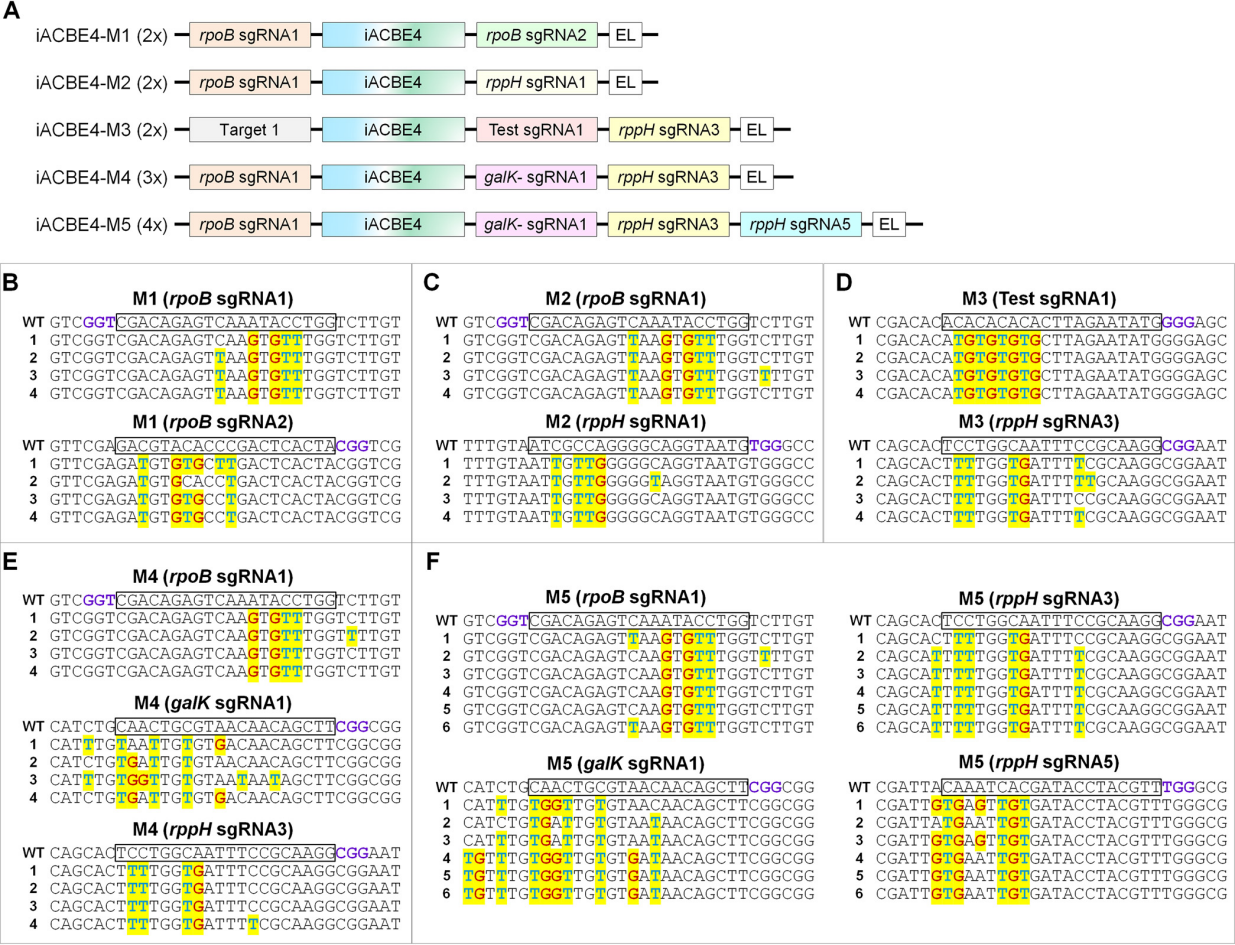
Evaluation of multiplex editing activities of iACBE4 in E. coli. (A) Architecture of plasmid constructs illustrating iACBE4 and tested sgRNAs. iACBE4 cassette comprises of pGlpT promoter and L3S2P21 terminator. The sgRNAs were expressed using pAtU6 promoter. (B to F) Sequence alignments of the targeted loci with multiple sgRNAs. The targeted sites from minimum four independent clones were sequenced and aligned. Protospacer sequence and PAM site are highlighted with box and in bold violet, respectively. WT indicates wild-type (native) DNA sequence. Clones are numbered at the left side; modified bases are highlighted in yellow; A-to-G changes shown in bold red and C-to-T in bold green. (B) Nucleobase conversion activities by iACBE4 at two target DNA sites in the same gene (*rpoB*) from E. coli genome. (C) Base conversion activities at two DNA sites located on different genes (*rpoB* and *rppH*) in the E. coli genome. (D) Base conversion activities by iACBE4 at DNA sites simultaneously targeting the plasmid (Target 1) and genomic sites (*rppH*) in the E. coli genome. (E and F) Nucleobases modified by iACBE4 in simultaneous targeting of 3× and 4× sgRNAs, respectively.

Multiplex editing includes simultaneous editing of two or more sites and offers a fast way of generating multisite mutants in a single generation. Multiplex editing with single-function BEs was achieved in several organisms, including bacteria and eukaryotes (16, 30, 31). We aimed to examine the potential of iACBE4 for concurrent base editing in multiple sites, which was not investigated in any of the previous ACBE studies. Five different multiplex ACBE systems (iACBE4-M1 to M5) were designed following a single-plasmid component system containing expression cassettes of BE fusions, sgRNAs, and target sites if needed (Fig. 3A). Dual base editing at two target DNA sites (2×) located on either the same gene (*rpoB* by iACBE4-M1, Fig. 3B); or two different genes (*rpoB* and *rppH* by iACBE4-M2) (Fig. 3C); or sites located on the plasmid and genomic site (T1 and *rppH* by iACBE4-M3, Fig. 3D); was successfully achieved in all the analyzed independent clones. In addition, simultaneous targeting of three genes (*galK*, *rpoB*, and *rppH*) with triple (3×) and quadruple (4×) sgRNAs assembled into iACBE4-M4 (Fig. 3E) and iACBE4-M5 (Fig. 3F), respectively, showed efficient co-editing of A/C positioned in targeted sites. Taken together, an optimized iACBE4 system could be utilized for efficient dual base multisite editing on the same or different genes in E. coli.

### Evaluation of PAM-relaxed BE-NG and iACBE-NG tools in bacteria.

The iACBE constructs include nCas9, which requires NGG as a PAM motif. Also, the targeted A/C must be available within the editable window, restricting the choice of targetable loci in the genome for broader use. Considering the strict requirement of PAM as one of the constraints for using nCas9-based BE and iACBE, we replaced nCas9 with nCas9-NG, a nickase form of PAM-relaxed Cas9-NG, which recognizes NGN as a PAM motif in the target sites (25). Analysis of two targets with NGG PAM (T1 and T2) by nCas9-NG derived ABEs (NG-ABE8e, NG-ABE9e) and CBEs (NG-PmCDA1, NG-evoCD1, NG-APOBEC3A) revealed efficient editing in editable windows thereby confirming the compatibility of BE tools with nCas9-NG (Fig. S2). Editing efficacies and editable windows of nCas9-NG-BEs (Fig. S2) were similar to corresponding nCas9-BEs (Fig. 1D), reaching up to 100% in editing windows. Besides, Cas9-NG reported to self-target the sgRNA-expression cassette, possibly raising the off-target issue by producing secondary sgRNAs (26). Native sgRNA scaffold begins with GTT immediately after the protospacer sequence. We found that all the Cas9-NG-BEs recognize GTT as a PAM motif in the sgRNA cassette and induced base editing in the narrower window and a relatively lower percentage than on-target editing (Fig. S2). Surprisingly, PmCDA1-derived NG-CBE showed no self-editing for both the tested sites similar to a recent report in plants (32). These results indicated that regardless of self-editing, nCas9-NG-based BEs enable base editing in E. coli.

10.1128/mbio.02296-22.2 FIG S2 PAM-flexible Cas9 (SpCas9-NG)-based editing by different single base editors and self-targeting in sgRNA region. (A) Two independent sgRNAs, including alternate Cs at even (Test sgRNA1) and odd (Test gRNA2) positions and vice versa in case of As with NGG as PAM used for SpCas9-NG nickase-based BE test. The self-targeting region is the sgRNA expression cassette consisting of a 20 bp protospacer sequence with native scaffold in plasmid vector. GTT is the start site of the native sgRNA scaffold presumably recognized as PAM by SpCas9-NG. (B to F) Base editing activities at on-target (Target 1 or 2) and self-target region (sgRNA expression cassette) by single-function base editors. The graph bar shows the mean of percentage values, and error bars indicate the standard error of the mean (mean ± SEM) of four independent biological replicates. Dots indicate the individual biological replicates. Download FIG S2, TIFF file, 1.5 MB. Copyright © 2023 Shelake et al. 2023 Shelake et al. https://creativecommons.org/licenses/by/4.0/ This content is distributed under the terms of the Creative Commons Attribution 4.0 International license.

**FIG 4:**
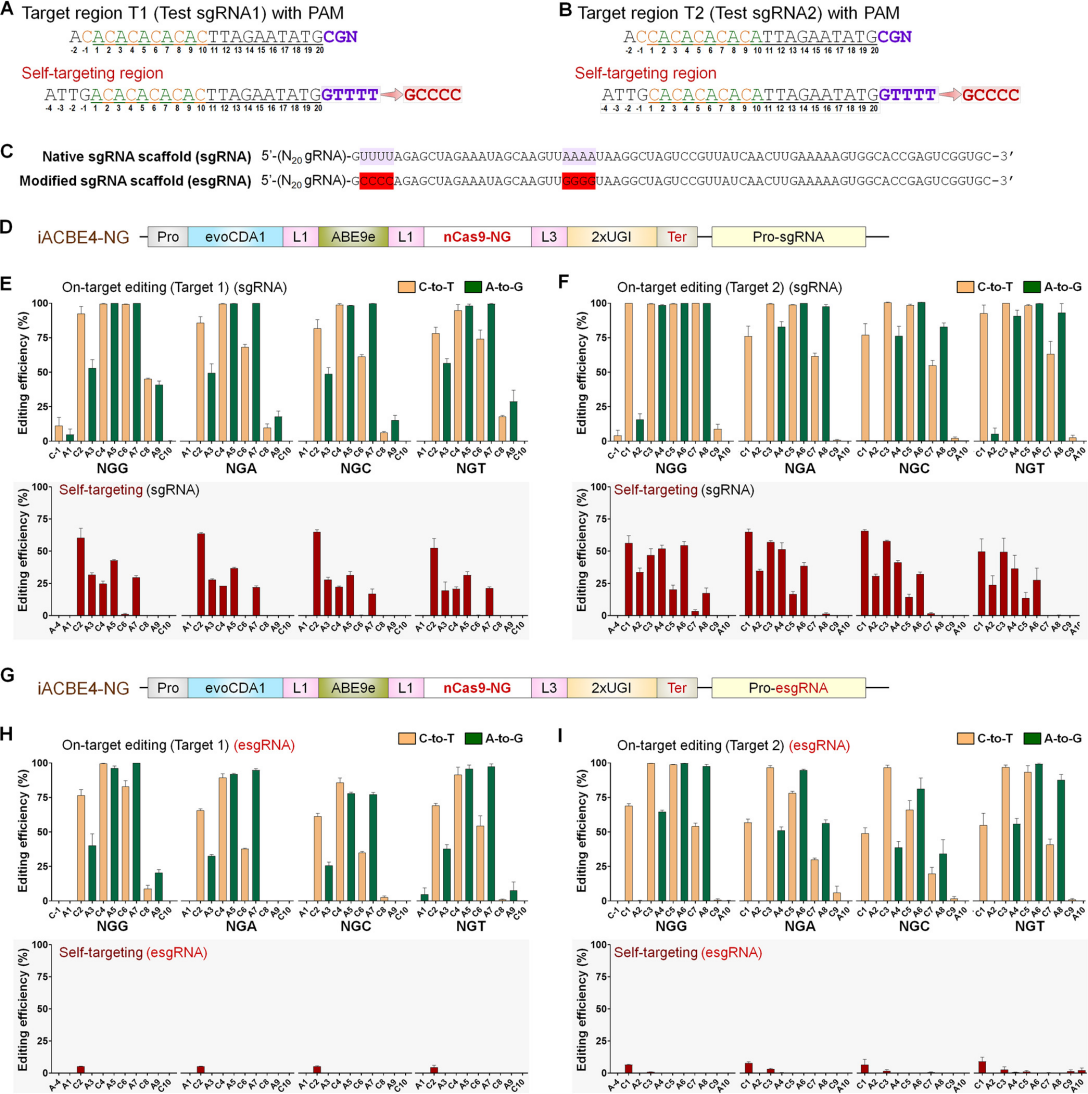
PAM-flexible SpCas9-NG nickase-based iACBE4-NG development for bacterial dual base editing. (A) The on-target site with protospacer adjacent motif (PAM) and self-targeting region in single guide RNA (sgRNA) cassette for Test sgRNA1 are shown. Editable As and Cs are highlighted in green and orange, respectively. The numbers are assigned by counting the distal base as 1 from the PAM, i.e., NGN counting as 21 to 23, where N is A/G/C/T. In the self-editing region, the native sgRNA scaffold starts with GTTTT (violet) and modified (esgRNA) starts with GCCCC (red). (B) The on-target and self-targeting regions of Test sgRNA2. (C) Nucleotide sequences of native and evolved sgRNAs. Nucleotides in native sgRNA colored in violet were changed in esgRNA (shaded in red). (D) Architecture of the plasmid construct illustrating iACBE4-NG and native sgRNA for testing editing features at NGN PAM motifs. L1, XTEN liner; L3, SGGS linker. (E and F) Concurrent A/C base editing activities by iACBE4-NG with native sgRNA cassettes at on-target and self-targeting regions. (G). Architecture of iACBE4-NG plasmid construct containing esgRNA. (H and I) On-target and self-target A/C base editing activities by iACBE4-NG consisting of esgRNA cassettes.

Inspired by the data of NG-BEs, we combined highly processive ABE8e-evoCDA1 and ABE9e-evoCDA1 with nCas9-NG to edit the NGN PAM sites generating iACBE2-NG and iACBE4-NG, respectively (Fig. 4; Table S2). We observed similar editing outcomes for iACBE2-NG (Fig. S3) and iACBE4-NG (Fig. 4) at T1 and T2 sites with the NGG motif, and hence, iACBE4-NG was pursued in further work. Target sites (T1 and T2) with NGN PAM (GGG, CGA, CGC, CGT) were cloned alongside desired ACBE components (Fig. 4A to D). To reduce self-editing in NG-based BEs, the modified sgRNA scaffold (esgRNA) was used (Fig. 4C) as described by Qin et al. (26) that preserved a high on-target indel generation rate while alleviating the self-editing in plants. At first, testing of iACBE4-NG with native sgRNA displayed higher co-editing of A/C at on-target and self-targeting regions in the T1 and T2 sites (Fig. 4E and F). For the T1 site, on-target editing against self-editing frequencies of positions 2 to 7 in the editable window with native sgRNA were 90.8% versus 30.8%, 83.8% versus 28.9%, 81.7% versus 26.9%, and 83% versus 23.1% at GGG, CGA, CGC, and CGT PAM motifs, respectively (Table S2). For T2 site, average on-target against self-editing frequencies of positions 1 to 8 with native sgRNA were 89.2% versus 35.6%, 77% versus 33.1%, 73.1% versus 30.4%, and 80.4% versus 25.1% at GGG, CGA, CGC, and CGT PAM motifs in the editable window, respectively (Table S2).

10.1128/mbio.02296-22.3 FIG S3 SpCas9-NG nickase based iACBE2 on-target and self-editing activities at tested synthetic target regions in Test sgRNA1 (panel A) and Test sgRNA2 (panel B). Download FIG S3, TIFF file, 1.6 MB. Copyright © 2023 Shelake et al. 2023 Shelake et al. https://creativecommons.org/licenses/by/4.0/ This content is distributed under the terms of the Creative Commons Attribution 4.0 International license.

10.1128/mbio.02296-22.6 TABLE S2 Average concurrent base editing activities induced by iACBE4-NG with native (sgRNA-GTTTT) and modified (sgRNA-GCCCC) sgRNA scaffold (esgRNA) at different A/C positions in the Target 1 (T1) and Target 2 (T2) sites. Average values were calculated from the editing percentage of four independent clones. Download Table S2, DOCX file, 0.05 MB. Copyright © 2023 Shelake et al. 2023 Shelake et al. https://creativecommons.org/licenses/by/4.0/ This content is distributed under the terms of the Creative Commons Attribution 4.0 International license.

The iACBE4-NG with esgRNA (Fig. 4G) revealed efficient on-target editing at the T1 (Fig. 4H) and T2 (Fig. 4I) sites, while self-editing was significantly reduced. For instance, average on-target and self-editing frequencies by iACBE4-NG with esgRNA for the T1 site were 82.5% versus 0.9%, 68.6% versus 0.9%, 60.5% versus 0.9%, and 74.3% versus 0.8% at GGG, CGA, CGC, and CGT PAM motifs, respectively (Table S2). Also, on-target against self-editing frequencies with esgRNA for the T2 site at GGG, CGA, CGC, and CGT PAM motifs were 72.9% versus 0.9%, 58% versus 1.4%, 48.2% versus 1.1%, and 66.1% versus 1.7%, respectively (Table S2). Overall, using esgRNA with iACBE4-NG could alleviate the self-targeting effect with comparable or slightly lower on-target editing for plasmid target sites in E. coli with the same editing features as native sgRNA (Fig. S4).

**FIG 5:**
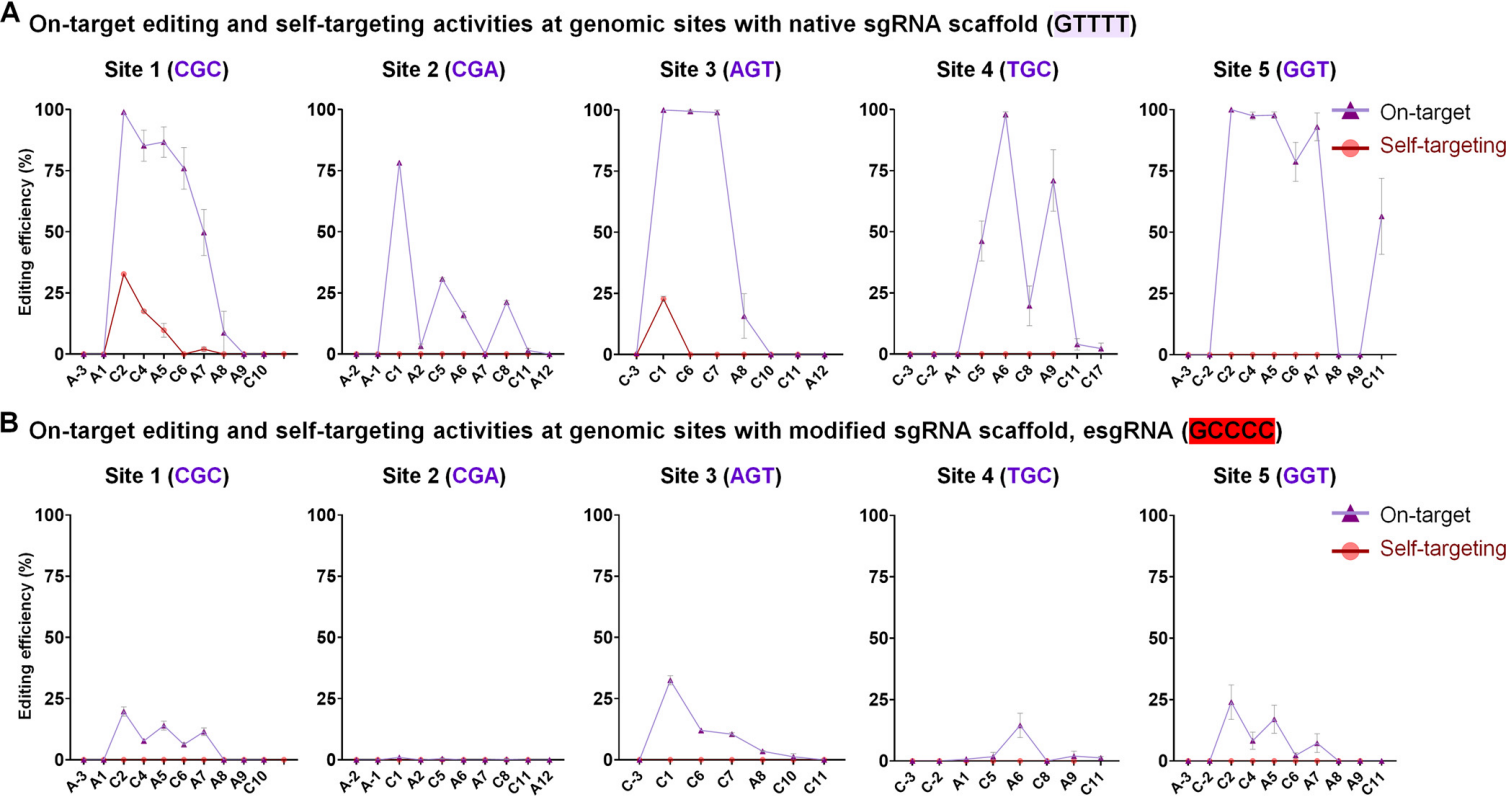
Modified sgRNA scaffold (esgRNA) reduces the editing efficiencies of iACBE4-NG at E. coli genomic target sites. (A) Analysis of on-target (violet) and self-target (red) editing activities at five E. coli genomic sites by iACBE-NG with native sgRNA scaffold starting with GTTTT. (B) On-target (violet) and self-target (red) editing outcomes at five E. coli genomic sites by iACBE-NG with modified sgRNA scaffold (esgRNA) starting with GCCCC.

Next, we applied the iACBE4-NG tool to edit the five genomic sites comprising native sgRNA and esgRNA with different NGN PAM motifs (Table S1). Among the analyzed genomic sites, there was no specific correlation between on-target and self-target editing frequencies by iACBE4-NG with native sgRNA scaffold (Fig. 5A). For instance, iACBE4-NG with native sgRNA displayed efficient editing at on-target (49.75% to 99% and 15.72% to 100%) and self-target (0% to 22% and 0% to 32%) regions for sites 1 and 3, respectively. At the same time, the iACBE4-NG construct showed high on-target editing but no self-targeting for sites 2, 4, and 5. Although we observed no self-editing in targeted genomic loci by iACBE4-NG with modified esgRNA, it also dramatically reduced the on-target A/C co-editing. All the five genomic sites by iACBE4-NG with esgRNA revealed a significant reduction in on-target editing activities (Fig. 5B).

Overall, iACBE-NG data indicate that the dual base editing tools comprising nCas9-NG and esgRNA are poorly compatible for modifying genomic loci, consistent with the recent ABE-NG reports in rice (33). Though the application of iACBE-NG greatly expands the choice of target sites to induce the dual base editing in E. coli, further research is needed to achieve high on-targeting with reduced or no self-editing with PAM-relaxed nucleases.

### Evolution of rifampicin-resistant protein (*rpoB*) of E. coli by iACBE systems.

**FIG 6:**
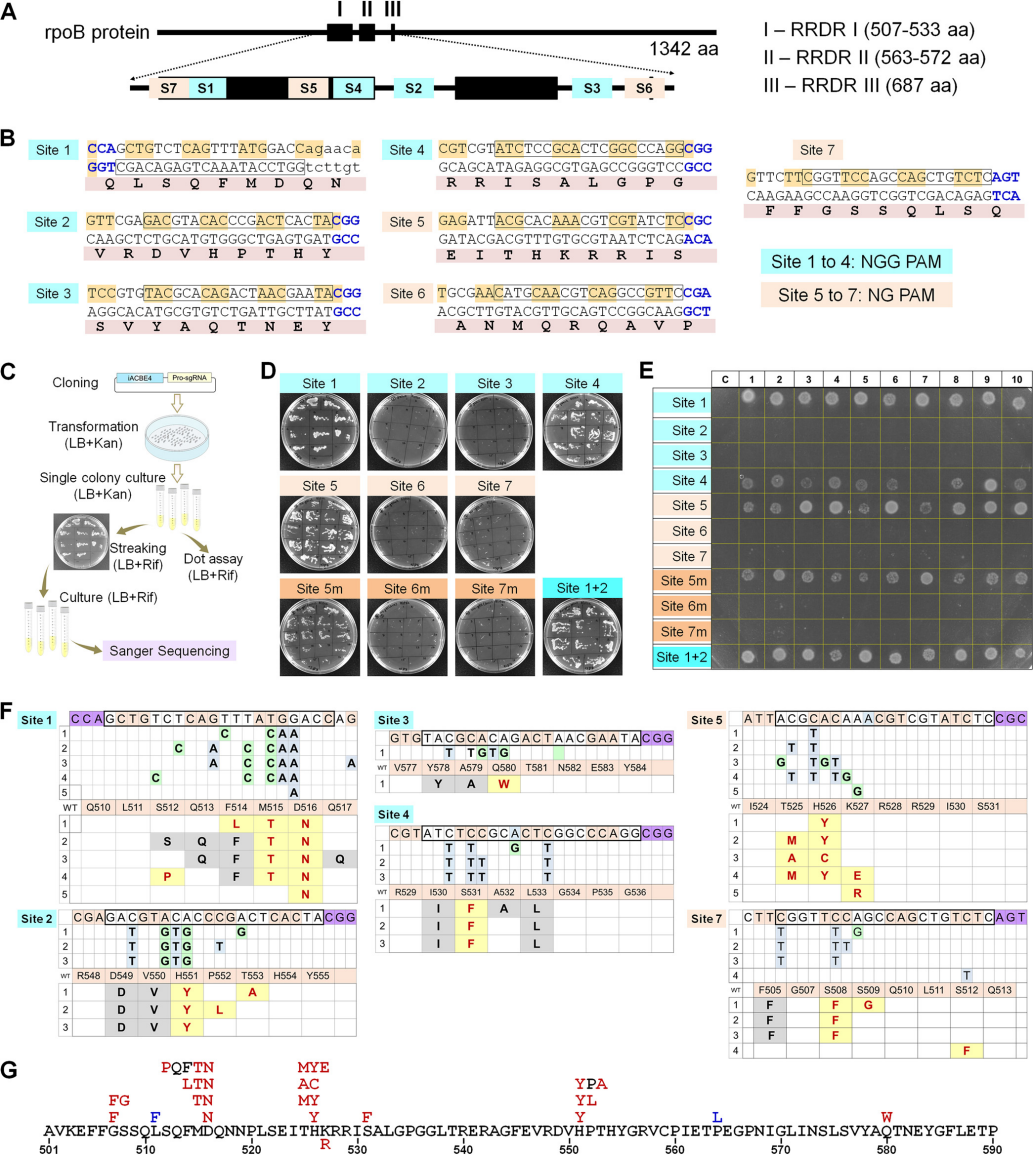
Targeted mutagenesis of the *rpoB* gene by iACBE4 and iACBE4-NG identifies novel (previously unknown) mutations bestowing rifampicin resistance in E. coli. (A) Outline of rifampicin resistance-determining regions (RRDRs) within rpoB protein (black boxes) and distribution of intended target sites (S1 to S7) for targeted mutagenesis. (B) The DNA and corresponding amino acid sequences of target sites. Sites 1 to 4 contained protospacers (highlighted in box) with NGG as PAM (blue) and were targeted by iACBE4. Protospacers at sites 5 to 7 contained NGN as PAM motifs. (C) Experimental setup for generating *rpoB* mutants and screening for rifampicin resistance (Rif^R^). (D and E) Screening of *rpoB* mutants performed by streak (D) and dot (E) assay on LB-agar plates with rifampicin. Cultures of individual clones streaked or dotted on plates were derived from the targeted mutagenesis by iACBE4 with native sgRNA scaffolds at sites 1 to 4. For sites 5 to 7, iACBE4-NG comprised of native scaffold (labeled as site 5, site 6, site 7) and modified (esgRNA) scaffold (labeled as site 5m, site 6m, site 7m). (F) Rifampicin-resistant *rpoB* alleles generated at different target sites. Multiple alleles with the same genotype are depicted only once, and nucleotide changes showing 20% or above are only considered in the analysis. WT indicates wild type amino acid sequence as a reference. Modified amino acids are highlighted in bold red with a yellow shade. Silent mutations showing editing at the DNA level but without altering amino acids are highlighted in a gray shade. (G) Distribution of mutations across 501 to 590 amino acid region of rpoB protein. Mutations found in the canonical BE window of iACBE are shown in red. Mutations found in the noncanonical iACBE window are shown in blue.

Engineered iACBE-based tools provide an excellent opportunity to mutate a gene of interest in native genetic background and simultaneously validate the gain-of-function. To demonstrate iACBE potential for *in situ* protein evolution studies, we chose the *rpoB* gene, which is implicated in rifampicin resistance (Rif^R^) in E. coli and several pathogenic microbes (34–38), suggestive of its clinical significance. The *rpoB* gene is highly conserved among bacteria and encodes the beta-subunit of DNA-dependent RNA polymerase. Several point (single amino-acid) mutations in RpoB are well-documented, conferring Rif^R^ in E. coli (35–38) (Fig. 6A; Table S3). The mode of action involves the transcription inhibition via rifampicin binding to the catalytic core of the RpoB enzyme. Three zones in RpoB are considered Rif^R^-determining region (RRDR) domains that include most of the known Rif^R^-related point mutations (37).

10.1128/mbio.02296-22.7 TABLE S3 The rifampicin resistance (Rif^R^) alleles mapped on the Rifampicin resistance determining region (RRDR) domains of *rpoB* gene in current and previous studies. Download Table S3, DOCX file, 0.05 MB. Copyright © 2023 Shelake et al. 2023 Shelake et al. https://creativecommons.org/licenses/by/4.0/ This content is distributed under the terms of the Creative Commons Attribution 4.0 International license.

The seven sites distributed across the three RRDR regions were randomly targeted (Fig. 6B). Sites 1 to 4 included protospacers with NGG PAM and sites 5 to 7 with NGN PAM. Seven independent iACBE4 constructs comprising desired nCas9 form (sites 1 to 4, nCas9; sites 5 to 7, nCas9-NG) were transformed into E. coli DH5-alpha strain and obtained clones were further screened for Rif^R^ (Fig. 6C). iACBE4-NG with native sgRNA or esgRNA were used for targeting sites 5 to 7. The multisite editing constructs comprising two (site 1 and 2) or three (site 1, 2, and 3) sgRNAs targeting the *rpoB* gene were also investigated, but clones mutated only in site 1 were recovered in the Rif^R^ screening, indicating mutants generated by multisite editing (at sites 2 and 3) were sensitive to rifampicin and did not survive. Single colony cultures were streaked or dotted on LB plates containing 50 μg/mL rifampicin to verify the Rif^R^ (Fig. 6D and E). The genotypes of independent Rif^R^ colonies that appeared on rifampicin-containing plates were determined by Sanger sequencing, and mutations with amino acid changes were mapped across the targeted sites. Among the tested regions, site 6 yielded no Rif^R^ clones. We identified a single or cluster of multiple amino acid substitutions responsible for Rif^R^ (Fig. 6F to G; Table S3). These results demonstrate the practical use of iACBE systems for saturated mutagenesis of the targeted genetic locus in E. coli cells, facilitating functional characterization of induced mutations thereof.

### Genome-wide off-target evaluation of the iACBE systems in E. coli.

**FIG 7:**
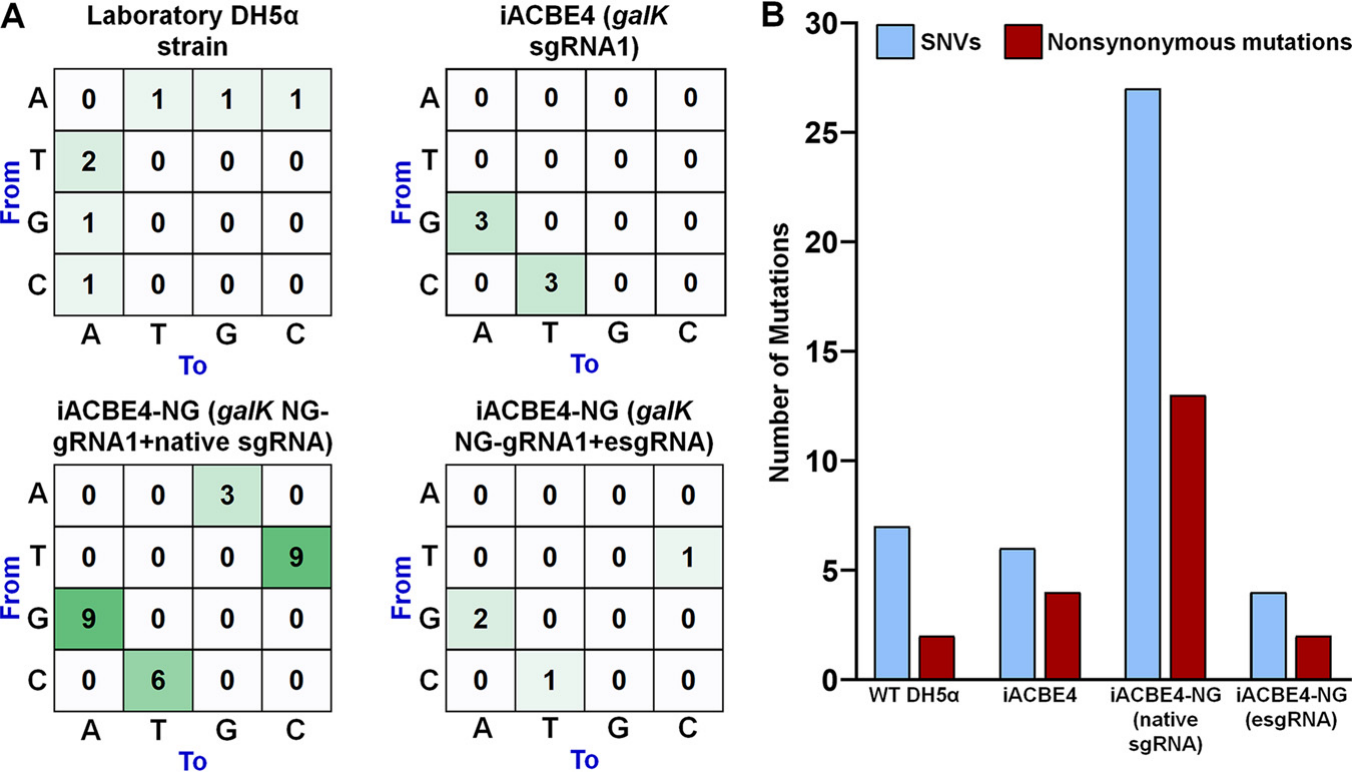
Genome-wide off-target analysis of the iACBE4 system in E. coli reveals a low level of sgRNA-independent mutations. (A) Distribution of the single nucleotide variations (SNVs) found in the full genome-sequenced laboratory DH5α strain and three edited clones (iACBE4 with *galK* gRNA1, iACBE4-NG with *galK* NG-gRNA1 containing native or esgRNA scaffold). The SNVs were calculated against the reference genome sequence GCA_022221385.1 for parental DH5α. The total number of SNVs in iACBE clones was recorded against the sequenced laboratory DH5α strain. The number in each cell represents the distribution of specific nucleotide base substitutions. A, adenine; T, thymine; G, guanine; C, cytosine. (B) The bar graph displays the total number of SNVs, and nonsynonymous (change of amino acid) mutations recorded as in panel A.

Precise editing without potential off-targets is critical for successfully applying CRISPR-based tools for genome engineering. To investigate potential off-targets induced by the iACBE systems, we carried out whole-genome sequencing (WGS) and unbiased profiling of single nucleotide variations (SNVs). One clone of each event of chromosome target (*galK*) editing from iACBE4 and iACBE4-NG with either native or esgRNA scaffold was analyzed using the WGS approach to assess the gRNA-independent SNVs (Fig. 7; Table S4). Additionally, WGS analysis of laboratory parental DH5α strain was performed to know the background noise of SNVs. We reasoned that aligning the WGS reads of each edited clone to the parental DH5α genome sequence allows a more precise evaluation of iACBE-specific off-targets, as performed previously (17, 30). SNV analysis revealed seven (laboratory DH5α strain), six (iACBE4 with *galK* gRNA1), 27 (iACBE4-NG-*galK* NG-gRNA1-native sgRNA), and four (iACBE4-NG-*galK* NG-gRNA1- esgRNA scaffold) off-target SNVs, resulting in two, four, 13, and two nonsynonymous mutations, respectively (Fig. 7; Table S4). These data are in line with previous reports on single BE-caused off-targets in other bacteria (17, 30). Therefore, genome-wide evaluation data indicate that our iACBE4 tool is a relatively safer GE system for E. coli.

10.1128/mbio.02296-22.8 TABLE S4 List of unique single nucleotide variations (SNV) detected in the analysis of whole-genome sequencing (WGS) data. Download Table S4, DOCX file, 0.05 MB. Copyright © 2023 Shelake et al. 2023 Shelake et al. https://creativecommons.org/licenses/by/4.0/ This content is distributed under the terms of the Creative Commons Attribution 4.0 International license.

## DISCUSSION

We engineered the dual base editing systems and applied for the first time in bacteria, employing the fusion of highly active ABE and CBE variants. Optimized iACBE toolset can be used for programmable dual base editing with high efficiencies at single or multisite targets in E. coli. The iACBE4-NG generated by using engineered Cas9-NG nickase (nCas9-NG), which recognizes the NGN PAM motif, further expands the target range of the iACBE toolset. In addition, characterized iACBE system exhibits high editing efficiencies up to 100%, which considerably cuts the workload of mutant verification. Unlike CRISPR/Cas9, which needs DSB generation, the engineered iACBE system is self-sufficient in introducing base conversion independent of NHEJ.

The iACBE-based screening of *rpoB* mutants related to Rif^R^ demonstrates its use in exploring the role of induced mutations in protein studies. In earlier studies, single amino-acid modifications in RpoB conferring Rif^R^ in E. coli are well-documented (35–38). We identified single and clusters of multiple amino-acid substitutions in RpoB imparting Rif^R^ (Table S3). For example, our data reveal nine Rif^R^ mutants with single amino-acid substitutions. Among those, five mutations overlapped with the previous reports (S512F, D516N, H526Y, S531F, P564L), and four mutations were unique (S508F, K527R, H551Y, Q580W). Additionally, nine clusters of multiple substitutions were identified, expanding the *rpoB* mutant library conferring Rif^R^ phenotype (Table S3), which may help to understand the structural aspects of antibiotic resistance. We note that the *rpoB* is an essential gene; therefore, it is possible that some of the editing events are actually not possible to be effectively selected during competitive growth between *rpoB* mutants against wild-type cells in the editing pool.

Recently, the ABE8e variant in ACBE design showed higher synchronized A/C editing efficiency in mammalian cells (10, 12), which is consistent with the iACBE data. Combining PAM-relaxed Cas9 versions and highly active BE deaminases broadens the scope using ACBE tools. PAM-relaxed Cas9 variants could be prone to two types of unwanted off-target effects. First, PAM-relaxed Cas9 could produce off-target editing at sgRNA-dependent loci. Second, sites recognizable by secondary sgRNAs created through self-editing increase off-targeting chances. Self-targeting is problematic due to the secondary gRNA production, but it may also reduce the on-target effect. Although there is no direct report about using the CRISPR tool based on Cas9-NG with esgRNA in bacteria, several studies in plants have evaluated the Cas9-NG-mediated indel generation and BE activities with esgRNA to alleviate the self-editing (26, 33, 39). The esgRNA may be compatible with Cas9-NG for generating indels, but it is poorly compatible with BE-NG and ACBE-NG. We found that the iACBE4-NG with esgRNA displayed similar on-target editing activities and decreased self-targeting on plasmid target sites. However, we observed that the esgRNA is not compatible with the iACBE tool for targeting genomic sites and dramatically reduced on-target efficiency.

In the case of the PmCDA1-based CBE-NG tool, we did not observe self-editing in bacteria which is consistent with the previous reports of PmCDA1-based CBEs with nCas9 (40), Cas9-NG, and SpRY (32) in plant systems. Hence, when desirable, we propose PmCDA1 as a first-choice CBE with PAM-relaxed Cas9 variants. Moreover, in the present and earlier studies, BE-NG- or iACBE4-NG-mediated editing was not diminished at estimated target sites. Therefore, despite the self-editing, BE and ACBE tools based on Cas9-NG may edit the sites with non-NGG PAM expanding the targeting scope of bacterial genome editing.

SpRY is another PAM-relaxed Cas9 variant that recognizes NYN (Y- C or T) and NRN (R- A or G) as PAM motifs (41). The sgRNAs targeting sites with NYN PAM motifs generated higher self-editing frequencies than on-target editing by SpRY in plants (42, 43). Also, combining esgRNA with SpRY is of no help because it causes a substantial reduction in on-target activities and higher self-editing in rice (44), which is an obvious outcome with GCC as PAM in esgRNA sequence. Therefore, we believe that the SpRY-mediated iACBE tools may induce higher self-editing in sgRNA cassettes than corresponding Cas9-NG-based BEs. Thus, it would be worthy of engineering specialized sgRNAs or Cas9 variants when implementing the PAM-relaxed BE and iACBE toolbox in bacteria and other organisms.

Prime editor (PE), another CRISPR-based GE tool (45), can theoretically introduce all kinds of base substitutions. The efficiency of PEs is low in different organisms, including E. coli (46), and predefined mutation sets can only be introduced. Therefore, better ACBEs that can induce the randomized combination of multiple base substitutions would be excellent endowments for protein evolution studies. Also, glycosylase base editor (CGBE), a new BE tool, introduces C-to-G, C-to-A, and C-to-T mutations depending on the DNA repair responses in different organisms (47–50). Incorporating CGBE into dual base editing systems would further extend the diversity of saturated mutagenesis, as described in a recent report (51). In the future, essential genes in their natural genomic context will be attractive targets by iACBE-mediated GE because of their importance in evolution, metabolic engineering, development of antibiotic resistance, and newer strain designs.

### Conclusions.

We developed iACBE, the first report of a dual base editing system for bacterial applications that provides a versatile tool for the induction of synchronized A-to-G and C-to-T mutations at the same target site. The successful application of iACBE4 for multiplex editing allowed concurrent diversification of multiple targets from plasmid or genomic sites. This study also reveals molecular insights into the compatibility of PAM-relaxed iACBE designs with native and esgRNA scaffolds for editing target sites with NGN PAM motifs. The iACBE toolset could be applied to introduce concurrent A/C base substitutions within the desired target genes in the genetic context facilitating functional analysis of constructed mutants in synthetic biology and basic microbiological research.

## MATERIALS AND METHODS

### E. coli strains and culture conditions.

The E. coli 10-beta strain was used for cloning of different biopart modules. The list of synthesized primers, generated plasmids, and biopart sequences used in the current study are provided in Table S5. Cloning and BE analyses were conducted in E. coli 10-beta and DH5α strains. The E. coli cells were grown in Luria-Bertani (LB) broth with suitable antibiotics.

10.1128/mbio.02296-22.9 TABLE S5 Primers, plasmids, and biopart sequences used for assembling dual base editing systems. Download Table S5, DOCX file, 0.1 MB. Copyright © 2023 Shelake et al. 2023 Shelake et al. https://creativecommons.org/licenses/by/4.0/ This content is distributed under the terms of the Creative Commons Attribution 4.0 International license.

E. coli strains were grown aerobically at 37°C in LB liquid medium (for 1 L, 5 g yeast extract, 10 g tryptone, and 10 g NaCl) or on LB-agar plates supplemented with desired antibiotics when required.

### Plasmid constructs, sgRNA designing, and cloning.

The necessary bioparts such as different Cas9 forms, deaminases, UGI, promoters, and terminators were amplified by traditional PCR employing a high-fidelity version of Phusion DNA polymerase (Thermo Fisher Scientific, Waltham, MA). Golden Gate assembly (52) and MoClo (53) kits were utilized for cloning various plasmid vectors, following the instructions provided in the kit protocols. Type IIS enzymes, BpiI and BsaI, were mainly used in the digestion-ligation process. BsaI and BpiI recognition sites were removed from bioparts for cloning in the Golden Gate system.

The pGlpT promoter and TerL3S2P21 terminator (24) were used to express single and dual base editing systems. The DNA sequences are provided in Table S5. The synthesized ABE8e (8-point mutations in ABE7.10) from Bioneer Co. (Daejon, South Korea) was used as a template in PCR to obtain the ABE9e (additional V82S/Q154R) variant by site-directed mutagenesis. Three CBE deaminase modules along with linker sequences were prepared by PCR using plasmid templates ordered from the Addgene, namely, PmCDA1 (Addgene #79620) (5), evoCDA1 (Addgene #122608) (21), and APOBEC3A (Addgene #119770) (27), respectively. The C-terminal PmCDA1-1xUGI was cloned with nCas9. The ABE8e or ABE9e with evoCDA1 or A3A, including the XTEN linker, were tethered at the N-terminal of nCas9. The 2xUGI module was prepared using (Addgene #122608) as a PCR template to fuse with the desired CBEs and iACBEs containing evoCDA1 and APOBEC3A.

The plant AtU6 promoter was amplified from (Addgene #46968) to express sgRNAs with the native (sgRNA) or modified (esgRNA) scaffold. The nCas9(D10A) and nCas9-NG(D10A) were cloned by the PCR-cloning strategy using template plasmids (Addgene #49771) and (Addgene #125616), respectively. Required sgRNA sequences with native or modified scaffold (esgRNA) were PCR amplified using a plasmid template (Addgene #46966) for further cloning and combined with pAtU6 for expression in E. coli cells. Target regions (T1 and T2) with appropriate PAM motifs were cloned into a universal target-acceptor plasmid (24) using the Type IIS BsmBI enzyme by digestion-ligation method.

### Bacterial transformation, plasmid isolation, and Sanger sequencing.

Cloning steps were performed by ligating the various biopart modules, followed by heat shock-mediated E. coli transformation. The 10-beta E. coli strains were used for cloning steps. Briefly, competent cells and digestion-ligation mix were exposed to heat shock at 45°C for 1 min, and 1 mL LB broth was added after incubating the tubes on ice for 3 min. Then, after shaking (180 rpm) the culture for 1 h at 37°C, it was spread on the LB agar (1.5%) plates containing appropriate antibiotics. Individual colonies appeared on LB-agar plates (with necessary antibiotics) after 18 h of incubation at 37°C were subjected to plasmid isolation and Sanger sequencing analysis at Cosmogentech Ltd. (Seoul, South Korea). For targeted mutagenesis assay by BE and iACBE systems, the E. coli cells were transformed with the desired plasmid vectors by heat shock method. Plasmid vectors consisting of BE or ACBE components without sgRNA-expression cassettes were used as control. As described earlier, independent clones from the LB-agar plate (with necessary antibiotics) were cultured in 3 mL antibiotic-containing LB media and allowed to grow at 37°C for a 24-h period. Plasmid DNA was purified for verification by restriction enzyme digestion, and sequencing analysis using Plasmid Mini-Prep Kit procured from BioFact Co. Ltd. (Daejeon, South Korea).

### Generation of *rpoB* mutant library and rifampicin resistance (Rif^R^) assay.

The desired sgRNA-expression cassettes were cloned together with iACBE4 or iACBE4-NG depending on the target site-PAM compositions in the *rpoB* gene. In particular, sites 1 to 4 consisted of sgRNA spacers with NGG and sites 5 to 7 with NGN PAM. Seven independent iACBE4 constructs were transformed into E. coli DH5-alpha strain and individual clones were grown on LB-agar plates containing kanamycin (50 μg/mL) were further screened for Rif^R^. Single colony cultures were streaked or dotted on LB plates containing 50 μg/mL rifampicin to verify the Rif^R^. Also, multisite editing iACBE4 constructs comprising two (site 1 and 2) or three (site 1, 2, and 3) sgRNAs were screened as described earlier. The genotypes of individual Rif^R^ colonies that grew on rifampicin-containing plates were determined by Sanger sequencing, and mutations with amino acid changes were mapped across the targeted sites.

### Analysis of base substitution activities.

To map the mutagenesis patterns in synthetic targets (T1 and T2) by Sanger sequencing, Plasmid Mini-Prep Kit was used for extracting plasmid DNA purchased from BioFact Co. Ltd. (Daejeon, South Korea). To investigate the mutations induced by different iACBE types at E. coli genomic sites, the colonies were randomly picked, cultured in LB broth with necessary antibiotics, and then incubated for 24 h at 180 rpm at 37°C. The genetic fragments were amplified using target region-specific oligos by PCR method and then subjected to Sanger sequencing. SnapGene software was used for Sanger data analysis (GSL Biotech; available at snapgene.com). The base editing efficiencies were estimated by the proportion of nonedited to mutated clones from the analyzed colonies. The frequency of base change (from C-to-T and A-to-G) was calculated using the EditR, an online base-editing analysis tool (28). The data were statistically analyzed and plotted in GraphPad Prism 9.3.1 (www.graphpad.com, last accessed on April 3, 2022).

### Genome-wide evaluation of off-targets of iACBE4 systems.

To analyze the off-target effects induced by the iACBE system, total genomic DNA was extracted from the laboratory DH5α strain and three edited clones (iACBE4 with *galK* gRNA1, iACBE4-NG with *galK* NG-gRNA1 containing native or esgRNA scaffold) using QIAamp DNA minikit (Qiagen, Germany). Whole-genome sequencing was performed at SEEDERS Inc. (SEEDERS, Daejeon, South Korea) with the Illumina NGS platform. Raw sequence reads were aligned to the reference E. coli genome (NCBI accession: GCA_022221385.1) or laboratory DH5α strain. The Illumina reads were preprocessed using Trimmomatic (v. 0.39) (54). SEEDERS in-house script (55) was utilized to map SNVs using BWA (0.7.17 r1188) and SAMtools (v 0.1.16) programs (56, 57). The alignment accuracy to the reference genome was 99.84% for all four samples. The SNV for a nucleobase was counted as homozygous (read rate 90%) or heterozygous (40%≤ read rate ≤60%) based on the read rate. Mutation calls found in all the samples and parent DH5α were excluded and not considered off-targets.

### Data availability.

The Illumina sequencing data generated for off-target evaluation has been deposited to NCBI: NCBI BioProject PRJNA906298; SRA accessions SRR22439416, SRR22452371, SRR22452578, and SRR22455127.

10.1128/mbio.02296-22.4 FIG S4 Summary of SpCas9-NG nickase-based (nSpCas9-NG) PAM-flexible iACBE-NG editing activities comprising native (GTTTT, sgRNA) and evolved (GCCCC, esgRNA) sgRNA scaffold. Synthetic Target 1 (T1) regions with variable PAM motifs were edited by iACBE4-NG and mean of percentage values of four independent biological replicates were plotted. (A) Comparison of individual A/C base editing at T1 at different NG PAMs by iACBE4-NG with native (GTTTT) and modified (GCCCC) sgRNA scaffold. (B) Combined summary of editing activities at all the tested PAMs. Download FIG S4, TIFF file, 1.2 MB. Copyright © 2023 Shelake et al. 2023 Shelake et al. https://creativecommons.org/licenses/by/4.0/ This content is distributed under the terms of the Creative Commons Attribution 4.0 International license.
