# Supplementary figures and images for: Improved Dual Base Editor Systems (iACBEs) for Simultaneous Conversion of Adenine and Cytosine in the Bacterium Escherichia coli
Source: mBio. 2023 Jan 10;14(1):e02296-22. doi: 10.1128/mbio.02296-22 (PMC9973308; doi:10.1128/mbio.02296-22)

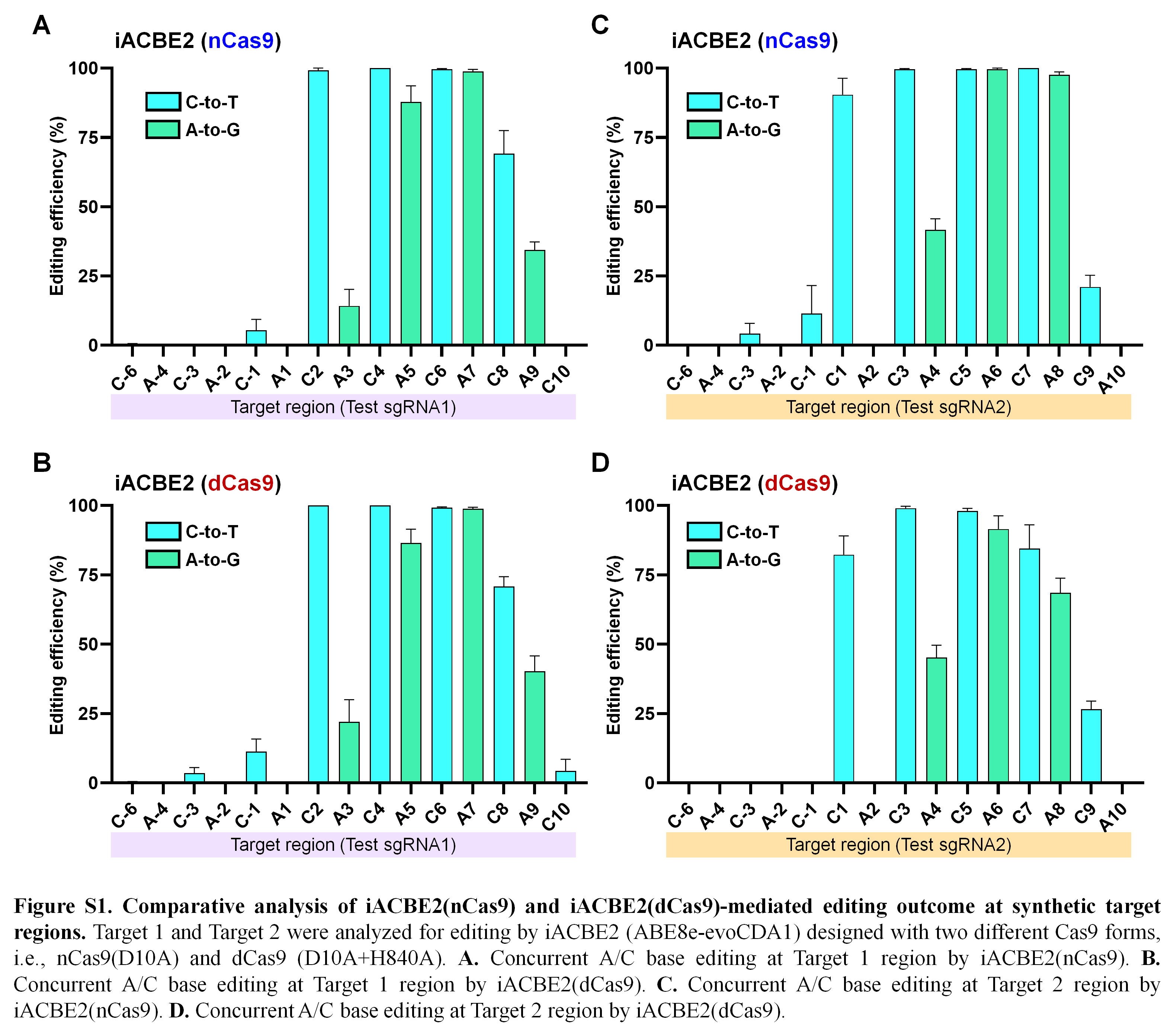

Supplement: FIG S1 [file mbio.02296-22-s0001.tif]

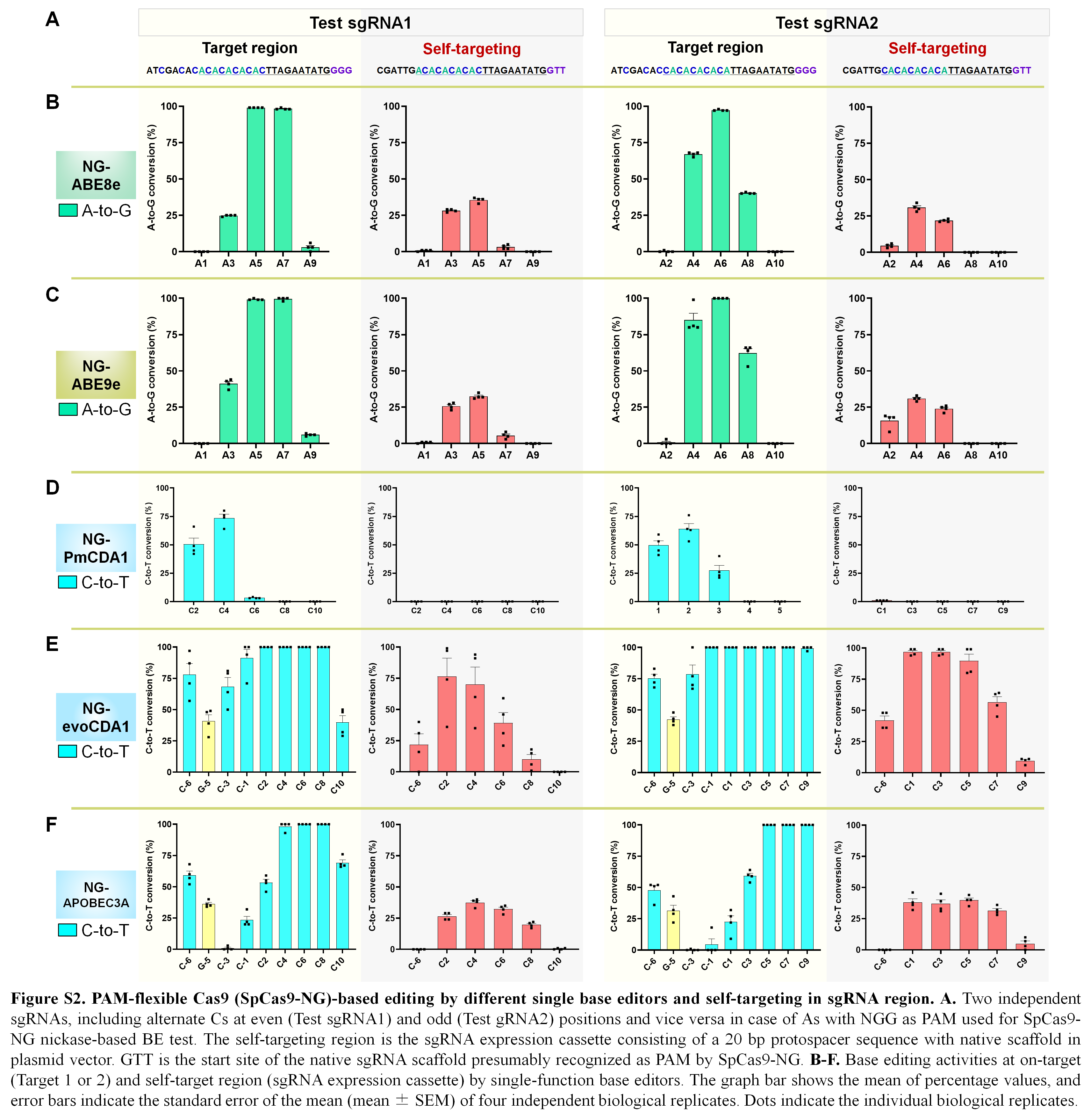

Supplement: FIG S2 [file mbio.02296-22-s0002.tif]

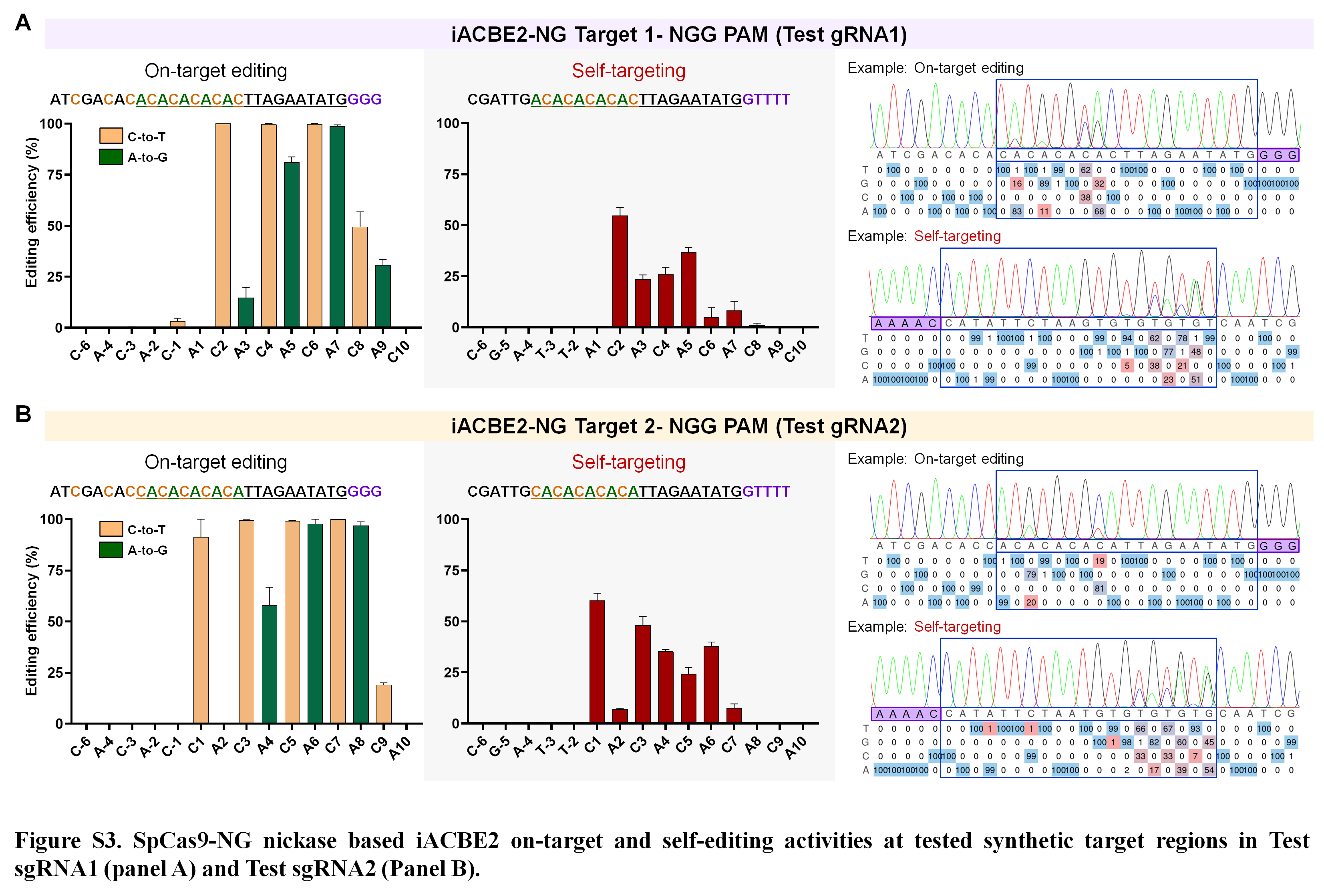

Supplement: FIG S3 [file mbio.02296-22-s0003.tif]

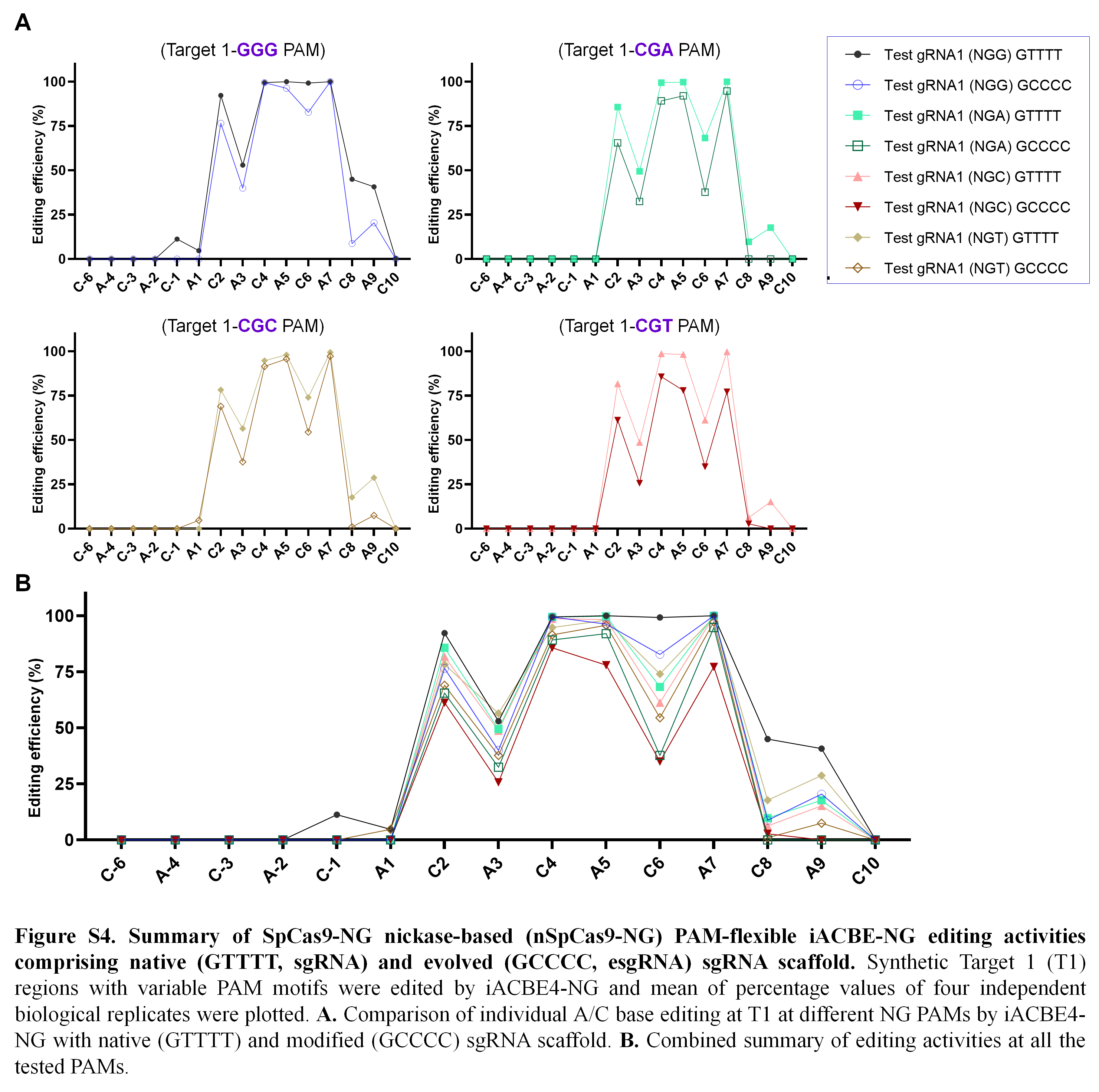

Supplement: FIG S4 [file mbio.02296-22-s0004.tif]
