# Supplementary material for: Improved Dual Base Editor Systems (iACBEs) for Simultaneous Conversion of Adenine and Cytosine in the Bacterium Escherichia coli
Source: mBio. 2023 Jan 10;14(1):e02296-22. doi: 10.1128/mbio.02296-22 (PMC9973308; doi:10.1128/mbio.02296-22)
Supplement: TABLE S1 [file mbio.02296-22-s0005.docx]

**Table S1.** Genomic target DNA sites with different Protospacer adjacent motifs (PAMs) analyzed for base conversion by iACBE4 and iACBE4-NG in Fig. 2 and Fig. 5, respectively.

| **Target site** | | **Protospacer sequence** | | | **PAM** | |
| --- | --- | --- | --- | --- | --- | --- |
| **Fig. 2** | | | | | | |
| *adhE* gRNA1 | | CCGAAAGCACACAGGGACTT | | | CGG | |
| *Pta* gRNA1 | | GCAAGAATCCAGCCCGCTGC | | | CGG | |
| *xlyB* gRNA1 | | CCCCACGCTTTCGCAACTTC | | | AGG | |
| *xlyB* gRNA2 | | TACGCCACACAATAATCCCC | | | AGG | |
| *rppH* gRNA1 | | ATCGCCAGGGGCAGGTAATG | | | TGG | |
| *rppH* gRNA2 | | AGGGGCAGGTAATGTGGGCC | | | CGG | |
| *rppH* gRNA3 | | TCCTGGCAATTTCCGCAAGG | | | CGG | |
| *rppH* gRNA4 | | CTACAAATTACCGAAACGTT | | | TGG | |
| *rppH* gRNA5 | | CAAATCACGATACCTACGTT | | | TGG | |
| *rpoB*-gRNA1 | | GGTCCATAAACTGAGACAGC | | | TGG | |
| *rpoB* gRNA2 | | GACGTACACCCGACTCACTA | | | CGG | |
| *rpoB* gRNA3 | | TACGCACAGACTAACGAATA | | | CGG | |
| *rpoB* gRNA4 | | ATCTCCGCACTCGGCCCAGG | | | CGG | |
| *galK* gRNA1 | | CAACTGCGTAACAACAGCTT | | | CGG | |
| **Fig. 5** | | | | | | |
| **Target site** | **Gene** | | **gRNA** | **Sequence** | | **PAM** |
| Site 1 | *rpoB* | | *rpoB* NG-gRNA1 | ACGCACAAACGTCGTATCTC | | CGC |
| Site 2 | *rpoB* | | *rpoB* NG-gRNA2 | CATGCAACGTCAGGCCGTTC | | CGA |
| Site 3 | *rpoB* | | *rpoB* NG-gRNA3 | CGGTTCCAGCCAGCTGTCTC | | AGT |
| Site 4 | *galK* | | *galK* NG-gRNA1 | ATTGCAGCAGCTTTATCATC | | TGC |
| Site 5 | *galK* | | *galK* NG-gRNA2 | GCGCACAAATCGCGCTTAAC | | GGT |
