# Supplementary material for: Improved Dual Base Editor Systems (iACBEs) for Simultaneous Conversion of Adenine and Cytosine in the Bacterium Escherichia coli
Source: mBio. 2023 Jan 10;14(1):e02296-22. doi: 10.1128/mbio.02296-22 (PMC9973308; doi:10.1128/mbio.02296-22)
Supplement: TABLE S2 [file mbio.02296-22-s0006.docx]

**Table S2.** Average concurrent base editing activities induced by iACBE4-NG with native (sgRNA-GTTTT) and modified (sgRNA-GCCCC) sgRNA scaffold (esgRNA) at different A/C positions in the Target 1 (T1) and Target 2 (T2) sites. Average values were calculated from the editing percentage of four independent clones.

| Target-PAM | Position of A/C | On-target base editing (%) | | | | Self-targeting (%) | | | |
| --- | --- | --- | --- | --- | --- | --- | --- | --- | --- |
|  |  | sgRNA-GTTTT | | esgRNA-GCCCC | | sgRNA-GTTTT | | esgRNA-GCCCC | |
|  |  | Average | Mean frequency | Average | Mean frequency | Average | Mean frequency | Average | Mean frequency |
| Target 1-NGG | C2 | 92.3 | 90.8 | 76.5 | 82.5 | 60.3 | 30.8 | 5.3 | 0.9 |
|  | A3 | 53.0 |  | 40.0 |  | 31.8 |  | 0.0 |  |
|  | C4 | 99.5 |  | 99.5 |  | 24.8 |  | 0.0 |  |
|  | A5 | 100.0 |  | 96.3 |  | 42.8 |  | 0.0 |  |
|  | C6 | 99.3 |  | 82.8 |  | 1.0 |  | 0.0 |  |
|  | A7 | 100.0 |  | 100.0 |  | 29.8 |  | 0.0 |  |
| Target 1-NGA | C2 | 85.8 | 83.8 | 65.5 | 68.6 | 63.8 | 28.9 | 5.3 | 0.9 |
|  | A3 | 49.5 |  | 32.5 |  | 28.0 |  | 0.0 |  |
|  | C4 | 99.5 |  | 89.3 |  | 23.0 |  | 0.0 |  |
|  | A5 | 99.8 |  | 92.0 |  | 36.8 |  | 0.0 |  |
|  | C6 | 68.3 |  | 37.8 |  | 0.0 |  | 0.0 |  |
|  | A7 | 100.0 |  | 94.8 |  | 22.0 |  | 0.0 |  |
| Target 1-NGC | C2 | 81.8 | 81.7 | 61.3 | 60.5 | 65.0 | 26.9 | 5.3 | 0.9 |
|  | A3 | 48.8 |  | 25.8 |  | 28.0 |  | 0.0 |  |
|  | C4 | 98.8 |  | 85.8 |  | 22.3 |  | 0.0 |  |
|  | A5 | 98.3 |  | 78.0 |  | 31.5 |  | 0.0 |  |
|  | C6 | 61.3 |  | 35.0 |  | 0.3 |  | 0.0 |  |
|  | A7 | 99.8 |  | 77.3 |  | 17.0 |  | 0.0 |  |
| Target 1-NGT | C2 | 78.3 | 83.0 | 69.0 | 74.3 | 52.5 | 23.1 | 4.5 | 0.8 |
|  | A3 | 56.5 |  | 37.8 |  | 19.5 |  | 0.0 |  |
|  | C4 | 94.8 |  | 91.5 |  | 20.8 |  | 0.0 |  |
|  | A5 | 98.0 |  | 95.8 |  | 31.5 |  | 0.0 |  |
|  | C6 | 74.0 |  | 54.5 |  | 0.3 |  | 0.0 |  |
|  | A7 | 99.5 |  | 97.3 |  | 21.3 |  | 0.0 |  |
| Target 2-NGG | C1 | 100.0 | 89.2 | 68.8 | 72.9 | 56.3 | 35.6 | 6.5 | 0.9 |
|  | A2 | 15.8 |  | 0.3 |  | 33.8 |  | 0.0 |  |
|  | C3 | 99.5 |  | 99.8 |  | 46.8 |  | 0.8 |  |
|  | A4 | 98.5 |  | 64.5 |  | 52.0 |  | 0.0 |  |
|  | C5 | 99.5 |  | 98.8 |  | 20.3 |  | 0.0 |  |
|  | A6 | 100.0 |  | 99.8 |  | 54.5 |  | 0.0 |  |
|  | C7 | 100.0 |  | 54.0 |  | 3.5 |  | 0.0 |  |
|  | A8 | 100.0 |  | 97.5 |  | 17.5 |  | 0.0 |  |
| Target 2-NGA | C1 | 76.0 | 77.0 | 56.8 | 58.0 | 65.0 | 33.1 | 7.8 | 1.4 |
|  | A2 | 0.0 |  | 0.0 |  | 34.8 |  | 0.0 |  |
|  | C3 | 99.5 |  | 96.8 |  | 57.0 |  | 3.3 |  |
|  | A4 | 83.0 |  | 51.0 |  | 51.5 |  | 0.3 |  |
|  | C5 | 98.8 |  | 78.3 |  | 16.8 |  | 0.0 |  |
|  | A6 | 100.0 |  | 94.8 |  | 38.5 |  | 0.0 |  |
|  | C7 | 61.5 |  | 30.0 |  | 0.0 |  | 0.0 |  |
|  | A8 | 97.5 |  | 56.3 |  | 1.3 |  | 0.0 |  |
| Target 2-NGC | C1 | 76.3 | 73.1 | 49.0 | 48.2 | 65.8 | 30.4 | 6.5 | 1.1 |
|  | A2 | 0.0 |  | 0.0 |  | 30.8 |  | 0.0 |  |
|  | C3 | 99.5 |  | 96.8 |  | 57.8 |  | 1.5 |  |
|  | A4 | 75.5 |  | 38.8 |  | 41.3 |  | 0.0 |  |
|  | C5 | 97.5 |  | 66.0 |  | 14.3 |  | 0.0 |  |
|  | A6 | 99.8 |  | 81.3 |  | 32.3 |  | 0.0 |  |
|  | C7 | 54.3 |  | 19.8 |  | 1.5 |  | 0.5 |  |
|  | A8 | 82.0 |  | 34.3 |  | 0.0 |  | 0.0 |  |
| Target 2-NGT | C1 | 92.8 | 80.4 | 54.8 | 66.1 | 49.5 | 25.1 | 9.0 | 1.7 |
|  | A2 | 5.3 |  | 0.0 |  | 23.8 |  | 0.0 |  |
|  | C3 | 100.0 |  | 97.0 |  | 49.3 |  | 2.8 |  |
|  | A4 | 90.8 |  | 55.8 |  | 36.5 |  | 0.5 |  |
|  | C5 | 98.3 |  | 93.3 |  | 13.5 |  | 1.0 |  |
|  | A6 | 99.8 |  | 99.3 |  | 27.8 |  | 0.0 |  |
|  | C7 | 63.3 |  | 40.8 |  | 0.0 |  | 0.3 |  |
|  | A8 | 93.0 |  | 87.8 |  | 0.3 |  | 0.0 |  |
