# Supplementary material for: Improved Dual Base Editor Systems (iACBEs) for Simultaneous Conversion of Adenine and Cytosine in the Bacterium Escherichia coli
Source: mBio. 2023 Jan 10;14(1):e02296-22. doi: 10.1128/mbio.02296-22 (PMC9973308; doi:10.1128/mbio.02296-22)
Supplement: TABLE S3 [file mbio.02296-22-s0007.docx]

**Table S3.** The rifampicin resistance (Rif^R^) alleles mapped on the Rifampicin resistance determining region (RRDR) domains of the *rpoB* gene in current and previous studies are listed. Novel Rif^R^ alleles found in the present work are shaded in yellow. Rif^R^ alleles reported in both present and prior studies are shaded in green.

| **Position** | **Amino acid** | **Allele genotype (DNA)** | **Amino acid substitution** | **Rif^R^** | **Reference** |
| --- | --- | --- | --- | --- | --- |
| 507 | G | GGT to GAT | G507D | Y | Garibyan et al., 2003 |
| 508 | S | TCC to CCC | S508P | Y | Garibyan et al., 2003 |
|  |  | TCC to TTC or TTT | S508F | Y | This study |
| 509 | S | AGC to CGC | S509R | Y | Garibyan et al., 2003 |
| 509+509 | SS | TCC-AGC to TTC-GGC | S508F+S509G | Y | This study |
| 511 | L | CTG to CCG | L511P | Y | Garibyan et al., 2003 |
|  |  | CTG to CAG | L511Q | Y | Garibyan et al., 2003 |
|  |  | CTG to AGG | L511R | Y | Garibyan et al., 2003 |
| 512 | S | TCT to ACT | S512P | Y | Severinov et al., 1993 |
|  |  | TCT to TTT | S512F | Y | Landick et al., 1990; this study |
|  |  | TCT to GCT | S512A | Y | Garibyan et al., 2003 |
|  |  | TCT to TAT | S512Y | Y | Garibyan et al., 2003 |
| 513 | Q | CAG to CGG | Q513R | Y | Severinov et al., 1993 |
|  |  | CAG to CTG | Q513L | Y | Jin and Gross, 1988 |
|  |  | CAG to CCG | Q513P | Y | Jin and Gross, 1988 |
|  |  | CAG to AAG | Q513K | Y | Garibyan et al., 2003 |
| 516 | D | GAC to AAC | D516N | Y | Ovchinnikov et al., 1981; this study |
|  |  | GAC to GCC | D516A | Y | Garibyan et al., 2003 |
|  |  | GAC to GGC | D516G | Y | Garibyan et al., 2003 |
|  |  | GAC to GTC | D516V | Y | Ovchinnikov et al., 1981 |
|  |  | GAC to TAC | D516Y | Y | Severinov et al., 1993 |
| 514+515+516 | FMD | TTT-ATG-GAC to CTT-ACA-AAC | F514L+M515T+D516N | Y | This study |
| 515+516 | MD | ATG-GAC to ACA-AAC | M515T+D516N | Y | This study |
| 512+515+516 | SQFMD | TCT-CAG-TTT-ATG-GAC to CCT-CAG-TTC-ACA-AAC | S512P+M515T+D516N | Y | This study |
| 518 | N | AAC to GAC | N518D | Y | Garibyan et al., 2003 |
| 522 |  | TCT to TTT | S522F | Y | Jin and Gross, 1988 |
|  |  | TCT to TAT | S522Y | Y | Garibyan et al., 2003 |
| 523 | E | GAG to GTG | E523V | Y | Garibyan et al., 2003 |
| 525 | T | ACG to AGG | T525R | Y | Garibyan et al., 2003 |
| 526 | H | CAC to TAC | H526Y | Y | Ovchinnikov et al., 1981; this study |
|  |  | CAC to CCC | H526P | Y | Severinov et al., 1993 |
|  |  | CAC to CGC | H526R | Y | Severinov et al., 1993 |
|  |  | CAC to CTC | H526L | Y | Garibyan et al., 2003 |
|  |  | CAC to AAC | H526N | Y | Garibyan et al., 2003 |
|  |  | CAC to CAA/CAG | H526Q | Y | Severinov et al., 1993 |
|  |  | CAC to GAC | H526D | Y | Garibyan et al., 2003 |
| 525+526 | TH | ACG-CAC to ATG-TAC | T525M+H526Y | Y | This study |
|  |  | ACG-CAC to GCG-TGT | T525A+H526C | Y | This study |
| 527 | K | AAA to AGA | K527R | Y | This study |
| 525+526+527 | THK | ACG-CAC-AAA to ATG-TAT-GAA | T525M+H526Y+K527E | Y | This study |
| 529 | R | CGT to TGT | R529C | Y | Jin and Gross, 1988 |
|  |  | CGT to CAT | R529H | Y | Severinov et al., 1993 |
|  |  | CGT to CTT | R529L | Y | Severinov et al., 1993 |
|  |  | CGT to TCT | R529S | Y | Jin and Gross, 1988 |
| 531 | S | TCC to TTC/TTT | S531F | Y | Ovchinnikov et al., 1981; this study |
|  |  | TCC to TAC | S531Y | Y | Severinov et al., 1993 |
| 532 | A | GCA to GTA | A532V | Y | Severinov et al., 1993 |
|  |  | GCA to GAA | A532E | Y | Severinov et al., 1993 |
| 533 | L | CTC to CCC | L533P | Y | Jin and Gross, 1988 |
|  |  | CTC to CAC | L533H | Y | Garibyan et al., 2003 |
|  |  | CTC to CGC | L533R | Y | Garibyan et al., 2003 |
| 534 | G | GGC to AGC | G534S | Y | Garibyan et al., 2003 |
|  |  | GGC to GAC | G534D | Y | Severinov et al., 1993 |
|  |  | GGC to TGC | G534C | Y | Garibyan et al., 2003 |
|  |  | GGC to GTC | G534V | Y | Garibyan et al., 2003 |
|  |  | GGC to GCC | G534A | Y | Garibyan et al., 2003 |
| 551 | H | CAC to TGC | H551Y | Y | This study |
| 551+552 | HP | CAC-CCG to TGC-CTG | H551Y+P552L | Y | This study |
| 551+553 | HPT | CAC-CCG-ACT to TGC-CCG-GCT | H551Y+T553A | Y | This study |
| 563 | T | ACC to CCC | T563P | Y | Jin and Gross, 1988 |
| 564 | P | CCT to CTT | P564L | Y | Ovchinnikov et al., 1981; this study |
|  |  | CCT to CGT | P564R | Y | Garibyan et al., 2003 |
| 570 | G | GGT to TGT | G570C | Y | Garibyan et al., 2003 |
| 572 | I | ATC to ACC | I572T | Y | Garibyan et al., 2003 |
|  |  | ATC to TTC | I572F | Y | Jin and Gross, 1988 |
|  |  | ATC to AAC | I572N | Y | Garibyan et al., 2003 |
|  |  | ATC to CTC | I572L | Y | Garibyan et al., 2003 |
|  |  | ATC to AGC | I572S | Y | Garibyan et al., 2003 |
|  |  | ATC to ATG | I572M | Y | Garibyan et al., 2003 |
| 574 | S | TCT to TTT | S574F | Y | Garibyan et al., 2003 |
|  |  | TCT to TAT | S574Y | Y | Garibyan et al., 2003 |
| 580 | Q | CAG TO TGG | Q580W | Y | This study |
| 687 | R | CGT to CAT | R687H | Y | Jin and Gross, 1988 |

Garibyan L, Huang T, Kim M, Wolff E, Nguyen A, Nguyen T, Diep A, Hu K, Iverson A, Yang H, Miller JH. 2003. Use of the *rpoB* gene to determine the specificity of base substitution mutations on the *Escherichia coli* chromosome. DNA Repair (Amst) 2:593–608.

Severinov K, Soushko M, Goldfarb A, Nikiforov V. 1993. Rifampicin region revisited. New rifampicin-resistant and streptolydigin-resistant mutants in the beta subunit of *Escherichia coli* RNA polymerase. J Biol Chem 268:14820–14825.

Landick R, Stewart J, Lee DN. 1990. Amino acid changes in conserved regions of the beta-subunit of *Escherichia coli* RNA polymerase alter transcription pausing and termination. Genes Dev 4:1623–1636.

Jin DJ, Gross CA. 1988. Mapping and sequencing of mutations in the *Escherichia coli* *rpoB* gene that lead to rifampicin resistance. J Mol Biol 202:45–58.

Ovchinnikov YuA, Monastyrskaya GS, Gubanov V V, Lipkin VM, Sverdlov ED, Kiver IF, Bass IA, Mindlin SZ, Danilevskaya ON, Khesin RB. 1981. Primary structure of *Escherichia coli* RNA polymerase nucleotide substitution in the beta subunit gene of the rifampicin resistant rpoB255 mutant. Mol Gen Genet 184:536–538.
