# Supplementary material for: Improved Dual Base Editor Systems (iACBEs) for Simultaneous Conversion of Adenine and Cytosine in the Bacterium Escherichia coli
Source: mBio. 2023 Jan 10;14(1):e02296-22. doi: 10.1128/mbio.02296-22 (PMC9973308; doi:10.1128/mbio.02296-22)
Supplement: TABLE S4 [file mbio.02296-22-s0008.docx]

**Table S4.** List of unique single nucleotide variations (SNV) detected in the analysis of whole genome sequencing (WGS) data. Raw sequence reads of the laboratory DH5α strain were aligned to the reference *E. coli* genome (NCBI accession: GCA_022221385.1), whereas iACBE4 strain data was aligned with the laboratory DH5α for mapping the SNVs. The SNV for a nucleobase was counted as homozygous (read rate 90%) or heterozygous (40%≤ read rate ≤60%) based on read rate. R- A or G, Y- C or T, W- A or T, M- A or C.

| **Strain** | **SNV** | **ID** | **Position** | **Protein** | **Reference** | **Allele** |
| --- | --- | --- | --- | --- | --- | --- |
| Laboratory DH5α Vs. NCBI reference strain | Homo | NZ_CP076470.1 | 61813 | YceD | T | A |
|  | Homo | NZ_CP076470.1 | 1050098 | Hypothetical protein | A | T |
|  | Homo | NZ_CP076470.1 | 3208426 | PTS glucose transporter subunit IIA | C | A |
|  | Hetero | NZ_CP076470.1 | 4112058 | Hypothetical protein | G | R |
|  | Hetero | NZ_CP076470.1 | 4112091 | Hypothetical protein | A | M |
|  | Hetero | NZ_CP076470.1 | 4112094 | Hypothetical protein | T | W |
|  | Homo | NZ_CP076470.1 | 4168921 | TonB-dependent receptor plug domain-containing protein | A | G |
| iACBE4 (galK sgRNA1) | Hetero | NZ_CP076470.1 | 1177703 | McrC | G | R |
|  | Hetero | NZ_CP076470.1 | 1177704 | McrC | G | R |
|  | Homo | NZ_CP076470.1 | 1234901 | FecA | C | T |
|  | Hetero | NZ_CP076470.1 | 1990061 | Glutathione S-transferase | C | Y |
|  | Hetero | NZ_CP076470.1 | 3091730 | HscB | C | Y |
|  | Hetero | NZ_CP076470.1 | 3997196 | tRNA-Val | G | R |
| iACBE4-NG (galK NG-gRNA1+native sgRNA) | homo | NZ_CP076470.1 | 248158 | Intergenic | T | C |
|  | homo | NZ_CP076470.1 | 541735 | MrdA | T | C |
|  | homo | NZ_CP076470.1 | 950210 | PcnB | C | T |
|  | homo | NZ_CP076470.1 | 970337 | Intergenic | G | A |
|  | homo | NZ_CP076470.1 | 995536 | HofC | G | A |
|  | homo | NZ_CP076470.1 | 1032304 | SgrT | G | A |
|  | homo | NZ_CP076470.1 | 1096256 | DnaK | G | A |
|  | homo | NZ_CP076470.1 | 1114568 | CreB | T | C |
|  | homo | NZ_CP076470.1 | 1382051 | L-methionine/branched-chain amino acid transporter | G | A |
|  | homo | NZ_CP076470.1 | 1455389 | Intergenic | G | A |
|  | homo | NZ_CP076470.1 | 1596818 | Acetylornithine deacetylase | G | A |
|  | homo | NZ_CP076470.1 | 1853169 | CbrC family protein | C | T |
|  | homo | NZ_CP076470.1 | 2224167 | IgaA | C | T |
|  | homo | NZ_CP076470.1 | 2445092 | DeaD | T | C |
|  | homo | NZ_CP076470.1 | 3119764 | GMP synthase | A | G |
|  | homo | NZ_CP076470.1 | 3169034 | DUF1176 domain-containing protein | G | A |
|  | homo | NZ_CP076470.1 | 3169037 | DUF1176 domain-containing protein | G | A |
|  | homo | NZ_CP076470.1 | 3344236 | NuoG | A | G |
|  | homo | NZ_CP076470.1 | 3416620 | YfaP family protein | T | C |
|  | homo | NZ_CP076470.1 | 3630186 | GalF | C | T |
|  | homo | NZ_CP076470.1 | 3752243 | Intergenic | C | T |
|  | homo | NZ_CP076470.1 | 4084897 | Dimethyl sulfoxide reductase subunit A | C | T |
|  | homo | NZ_CP076470.1 | 4206714 | Nitrate reductase subunit beta | C | T |
|  | homo | NZ_CP076470.1 | 4367417 | Kojibiose phosphorylase | T | C |
|  | hetero | NZ_CP076470.1 | 4367422 | Kojibiose phosphorylase | T | Y |
|  | homo | NZ_CP076470.1 | 4370980 | Zinc-binding alcohol dehydrogenase | A | G |
|  | homo | NZ_CP076470.1 | 4496637 | Bifunctional acetaldehyde-CoA/alcohol dehydrogenase | T | C |
| iACBE4-NG (galK NG-gRNA1+esgRNA) | homo | NZ_CP076470.1 | 168370 | Bifunctional acid phosphatase/4-phytase | G | A |
|  | hetero | NZ_CP076470.1 | 386550 | ClsB | G | R |
|  | homo | NZ_CP076470.1 | 3401680 | YfaL/EhaC | C | T |
|  | homo | NZ_CP076470.1 | 4367417 | Kojibiose phosphorylase | T | C |
