# Supplementary material for: Improved Dual Base Editor Systems (iACBEs) for Simultaneous Conversion of Adenine and Cytosine in the Bacterium Escherichia coli
Source: mBio. 2023 Jan 10;14(1):e02296-22. doi: 10.1128/mbio.02296-22 (PMC9973308; doi:10.1128/mbio.02296-22)
Supplement: TABLE S5 [file mbio.02296-22-s0009.docx]

**Table S5.** Primers, plasmids and biopart sequences used for assembling dual base editing systems.

| **Name** | **Sequence (5’-3’)** | **Description** |
| --- | --- | --- |
| **Cloning of sgRNAs with native scaffold** | | |
| Test gRNA1-F | TGTGGTCTCAATTGACACACACACTTAGAATCTGGTTTTAGAGCTAGAAATAGCAAGTTAAAAT | sgRNA PCR and cloning |
| Test gRNA2-F | TGTGGTCTCAATTGCACACACACATTAGAATCTGGTTTTAGAGCTAGAAATAGCAAGTTAAAAT | sgRNA PCR and cloning |
| *adhE* gRNA1-F | TGTGGTCTCAATTGCCGAAAGCACACAGGGACTTGTTTTAGAGCTAGAAATAGC | sgRNA PCR and cloning |
| *Pta* gRNA1-F | TGTGGTCTCAATTGGCAAGAATCCAGCCCGCTGCGTTTTAGAGCTAGAAATAGC | sgRNA PCR and cloning |
| *xlyB* gRNA1-F | TGTGGTCTCAATTGCCCCACGCTTTCGCAACTTCGTTTTAGAGCTAGAAATAGC | sgRNA PCR and cloning |
| *xlyB* gRNA2-F | TGTGGTCTCAATTGTACGCCACACAATAATCCCCGTTTTAGAGCTAGAAATAGC | sgRNA PCR and cloning |
| *rppH* gRNA1-F | TGTGGTCTCAATTGATCGCCAGGGGCAGGTAATGGTTTTAGAGCTAGAAATAGCAAGTTAAAAT | sgRNA PCR and cloning |
| *rppH* gRNA2-F | TGTGGTCTCAATTGAGGGGCAGGTAATGTGGGCCGTTTTAGAGCTAGAAATAGCAAGTTAAAAT | sgRNA PCR and cloning |
| *rppH* gRNA3-F | TGTGGTCTCAATTGTCCTGGCAATTTCCGCAAGGGTTTTAGAGCTAGAAATAGCAAGTTAAAAT | sgRNA PCR and cloning |
| *rppH* gRNA4-F | TGTGGTCTCAATTGCTACAAATTACCGAAACGTTGTTTTAGAGCTAGAAATAGCAAGTTAAAAT | sgRNA PCR and cloning |
| *rppH* gRNA5-F | TGTGGTCTCAATTGCAAATCACGATACCTACGTTGTTTTAGAGCTAGAAATAGCAAGTTAAAAT | sgRNA PCR and cloning |
| *rpoB* gRNA1-F | TGTGGTCTCAATTGGGTCCATAAACTGAGACAGCGTTTTAGAGCTAGAAATAGCAAGTTAAAAT | sgRNA PCR and cloning |
| *rpoB* gRNA2-F | TGTGGTCTCAATTGGACGTACACCCGACTCACTAGTTTTAGAGCTAGAAATAGCAAGTTAAAAT | sgRNA PCR and cloning |
| *rpoB* gRNA3-F | TGTGGTCTCAATTGTACGCACAGACTAACGAATAGTTTTAGAGCTAGAAATAGCAAGTTAAAAT | sgRNA PCR and cloning |
| *rpoB* gRNA4-F | TGTGGTCTCAATTGATCTCCGCACTCGGCCCAGGGTTTTAGAGCTAGAAATAGC | sgRNA PCR and cloning |
| *galK* gRNA1-F | TGTGGTCTCAATTGCAACTGCGTAACAACAGCTTGTTTTAGAGCTAGAAATAGCAAGTTAAAAT | sgRNA PCR and cloning |
| sgRNA Rev | TGTGGTCTCAAGCGTAATGCCAACTTTGTAC | sgRNA PCR and cloning |
| *rpoB* NG-sgRNA1 | TGTGGTCTCAATTGACGCACAAACGTCGTATCTCGTTTTAGAGCTAGAAATAGC | PCR and cloning of sgRNA |
| *rpoB* NG-sgRNA2 | TGTGGTCTCAATTGCATGCAACGTCAGGCCGTTCGTTTTAGAGCTAGAAATAGC | PCR and cloning of sgRNA |
| *rpoB* NG-sgRNA3 | TGTGGTCTCAATTGCGGTTCCAGCCAGCTGTCTCGTTTTAGAGCTAGAAATAGC | PCR and cloning of sgRNA |
| *galK* NG-sgRNA1 | TGTGGTCTCAATTGATTGCAGCAGCTTTATCATCGTTTTAGAGCTAGAAATAGC | PCR and cloning of sgRNA |
| *galK* NG-sgRNA2 | TGTGGTCTCAATTGGCGCACAAATCGCGCTTAACGTTTTAGAGCTAGAAATAGC | PCR and cloning of sgRNA |
| **Cloning of sgRNAs with evolved scaffold (esgRNA)** | | |
| Test esgRNA1-F | TGTGGTCTCAATTGACACACACACTTAGAATATGGCCCCAGAGCTAGAAATAGCAAGTTGGGGTAAGGCTAGTCCGTTATC | PCR and cloning of Test sgRNA1 with esgRNA |
| Test esgRNA2-F | TGTGGTCTCAATTGCACACACACATTAGAATATGGCCCCAGAGCTAGAAATAGCAAGTTGGGGTAAGGCTAGTCCGTTATC | PCR and cloning of Test sgRNA2 with esgRNA |
| *rpoB* NG-esgRNA1 | TGTGGTCTCAATTGACGCACAAACGTCGTATCTCGCCCCAGAGCTAGAAATAGCAAGT | PCR and cloning of sgRNA with esgRNA |
| *rpoB* NG-esgRNA2 | TGTGGTCTCAATTGCATGCAACGTCAGGCCGTTCGCCCCAGAGCTAGAAATAGCAAGT | PCR and cloning of sgRNA with esgRNA |
| *rpoB* NG-esgRNA3 | TGTGGTCTCAATTGCGGTTCCAGCCAGCTGTCTCGCCCCAGAGCTAGAAATAGCAAGT | PCR and cloning of sgRNA with esgRNA |
| *galK* NG-esgRNA1 | TGTGGTCTCAATTGATTGCAGCAGCTTTATCATCGCCCCAGAGCTAGAAATAGCAAGT | sgRNA with esgRNA PCR and cloning |
| *galK* NG-esgRNA2 | TGTGGTCTCAATTGGCGCACAAATCGCGCTTAACGCCCCAGAGCTAGAAATAGCAAGT | PCR and cloning of sgRNA with esgRNA |
| **Target oligos** | | |
| T1-NGG (Test gRNA1)-F | CAGTCCGTCTCTTTCGTCAAGTGCGACTCCCCATCGACACACACACACACTTAGAATATGGGGAGCTTTGAGACGGACT | Target 1-NGG (Test sgRNA1) |
| T1-NGG (Test gRNA1)-R | AGTCCGTCTCAAAGCTCCCCATATTCTAAGTGTGTGTGTGTGTCGATGGGGAGTCGCACTTGACGAAAGAGACGGACTG | Target 1-NGG (Test sgRNA1) |
| T2-NGG (Test gRNA2)-F | CAGTCCGTCTCTTTCGTCAAGTGCGACTCCCCATCGACACCACACACACATTAGAATCTGGGGAGCTTTGAGACGGACT | Target 2-NGG (Test sgRNA2) |
| T2-NGG (Test gRNA2)-R | AGTCCGTCTCAAAGCTCCCCAGATTCTAATGTGTGTGTGGTGTCGATGGGGAGTCGCACTTGACGAAAGAGACGGACTG | Target 2-NGG (Test sgRNA2) |
| T1-NGA (Test gRNA1)-F | CAGTCCGTCTCTTTCGCCCCATCGACACACACACACACTTAGAATATGCGAAGCTTTGAGACGGACT | Target 1-NGA (Test sgRNA1) |
| T1-NGA (Test gRNA1)-R | AGTCCGTCTCAAAGCTTCGCATATTCTAAGTGTGTGTGTGTGTCGATGGGGCGAAAGAGACGGACTG | Target 1-NGA (Test sgRNA1) |
| T1-NGC (Test gRNA1)-F | CAGTCCGTCTCTTTCGCCCCATCGACACACACACACACTTAGAATATGCGCAGCTTTGAGACGGACT | Target 1-NGC (Test sgRNA1) |
| T1-NGC (Test gRNA1)-R | AGTCCGTCTCAAAGCTGCGCATATTCTAAGTGTGTGTGTGTGTCGATGGGGCGAAAGAGACGGACTG | Target 1-NGC (Test sgRNA1) |
| T1-NGT (Test gRNA1)-F | CAGTCCGTCTCTTTCGCCCCATCGACACACACACACACTTAGAATATGCGTAGCTTTGAGACGGACT | Target 1-NGT (Test sgRNA1) |
| T1-NGT (Test gRNA1)-R | AGTCCGTCTCAAAGCTACGCATATTCTAAGTGTGTGTGTGTGTCGATGGGGCGAAAGAGACGGACTG | Target 1-NGT (Test sgRNA1) |
| T2-NGA (Test gRNA2)-F | CAGTCCGTCTCTTTCGCCCCATCGACACCACACACACATTAGAATATGCGAAGCTTTGAGACGGACT | Target 2-NGA (Test sgRNA2) |
| T2-NGA (Test gRNA2)-R | AGTCCGTCTCAAAGCTTCGCATATTCTAATGTGTGTGTGGTGTCGATGGGGCGAAAGAGACGGACTG | Target 2-NGA (Test sgRNA2) |
| T2-NGC (Test gRNA2)-F | CAGTCCGTCTCTTTCGCCCCATCGACACCACACACACATTAGAATATGCGCAGCTTTGAGACGGACT | Target 2-NGC (Test sgRNA2) |
| T2-NGC (Test gRNA2)-R | AGTCCGTCTCAAAGCTGCGCATATTCTAATGTGTGTGTGGTGTCGATGGGGCGAAAGAGACGGACTG | Target 2-NGC (Test sgRNA2) |
| T2-NGT (Test gRNA2)-F | CAGTCCGTCTCTTTCGCCCCATCGACACCACACACACATTAGAATATGCGTAGCTTTGAGACGGACT | Target 2-NGT (Test sgRNA2) |
| T2-NGT (Test gRNA2)-R | AGTCCGTCTCAAAGCTACGCATATTCTAATGTGTGTGTGGTGTCGATGGGGCGAAAGAGACGGACTG | Target 2-NGT (Test sgRNA2) |
| **Target amplification and Sanger Sequencing** | | |
| L1-F1 | GATGGGCTGCCTGTATCGAGT | Target 1 and 2 region seq |
| adh-F | GGATATCCGTAAACGTAT | *adhE* PCR |
| adh-R | CGGGTTGTCGTTCGCATT | *adhE* PCR |
| Pta-F | GTAAGCACCAGTTTGCCCAG | *Pta* PCR and Sanger seq. |
| Pta-R | CCATTTCGTAACCGCCAGTC | *Pta* PCR |
| xly-F | GCCAGCGATATGTCTGAC | *xlyB* PCR |
| xly-R | GCTGATATCCGCCAGCATC | *xlyB* PCR |
| rppH-F | CGGCTATCCACCCCTTCCTCTG | *rppH* PCR |
| rppH-R | GATTTCTGCATCGCCGCTCACC | *rppH* PCR |
| rppH-Seq F1 | CTCTGCACATAACTGTGAG | *rppH* Sanger seq. |
| rpoB-F | CCTCGGCAACCGTCGTATCC | *rpoB* PCR |
| rpoB-R1 | CCTGGGCGATAACGTAGTTGC | *rpoB* PCR |
| rpoB-R2 | GGATACATCTCGTCTTCG | *rpoB* PCR |
| rpoB-Seq F1 | GCGGAAAACCAGTTCCGCG | *rpoB* Sanger seq. |
| galK-F | GCCAACGCATTTGGCTACCCTG | *galK* PCR |
| galK-R | CATGCGCAACAGCGTTGAACTC | *galK* PCR |
| galK-Seq F1 | GGTGAACACACCGACTAC | *galK* Sanger seq. |
| Self-edit F | CTCGGTACCAAATTCCAG | sgRNA cassette Sanger seq. |
| Self-edit R | GCACATACAAATGGACGAACGGAT | sgRNA cassette Sanger seq. |
| **Biopart PCR for cloning** | | |
| pGlpT-F1 | GAGAAGACTTGGAGGAAAGTGAAACGTGATTTCATGCGTC | pGlpT (Pro-5U) PCR |
| pGlpT-R1 | ACTGAAGACAACATTCTAGTATTTCTCCTCTTTCTCTAG | pGlpT (Pro-5U) PCR |
| pGlpT-R2 | ACTGAAGACAAATGGCTAGTATTTCTCCTCTTTCTCTAG | pGlpT (Pro-5Uf) PCR |
| L3S2P2-F1 | GAGAAGACTTGCTTCTCGGTACCAAATTCCAGAAAAGAGGCCTCCCGAAAGGGGGGCCTTTTTTCGTTTTGGTCCCGCTAAGTCTTCACT | TerL3S2P21 (3U-Ter) cloning |
| L3S2P2-R1 | AGTGAAGACTTAGCGGGACCAAAACGAAAAAAGGCCCCCCTTTCGGGAGGCCTCTTTTCTGGAATTTGGTACCGAGAAGCAAGTCTTCTC | TerL3S2P21 (3U-Ter) cloning |
| eCDA-F1 | GCATGAAGACTTCCATGAAACGGACAGCCGACGGAAGCGAGTTCGAGTCA | evoCDA domestication (NT1) |
| eCDA-R1 | CAGTGAAGACTTGCTCTCTTCAGTGTTTTCTCCAGCCACCGATTC | evoCDA domestication (NT1) |
| eCDA-F2 | GCATGAAGACTTGAGCCGAGAAGCGGAGAAGCGA | evoCDA domestication (NT1) |
| eCDA-R2 | CAGTGAAGACTTCATTCCGCTGCCGCCGCTGCTGCCGCCACT | evoCDA domestication (NT1) |
| 2xU-F1 | GCATGAAGACTTGCTTCTAGCGGGGGGAGCACTAATCTGAGCGACA | 2xUGI cloning |
| 2xU-R1 | CAGTGAAGACTTTACCTTAGACTTTCCTCTTCTTCTTGGGCTCGA | 2xUGI cloning |
| A3A-F1 | GCATGAAGACTTCCATGAAACGGACAGCCGACGGAAGCGAGTTCGAGTCACCAAAGAAGAAGCGGAAAGTCGAGGCCAGCCCGGCTAGCGGCCCAAGG | APOBEC3A cloning (NT1) |
| A3A-R1 | CAGTGAAGACTTCATTCCCTTAAGAGATTCTGGGGTGGCCGAC | APOBEC3A cloning (NT1) |
| nCas9-NG-F1 | ACTGAAGACTTAATGGACAAGAAGTACAGCATCGGCCTG | nCas9-NG cloning (CDS1ns) |
| nCas9-NG-F2 | ACTGAAGACTTAGGTATGGACAAGAAGTACAGCATCGGCCTG | nCas9-NG cloning (CDS2ns) |
| nCas9-NG-F1 | CAGTGAAGACTTCGAACCCCCGCTCCCGCCGCTCCCGCCGCTGTCACCTCCC | nCas9-NG cloning (CDS1ns/CDS2ns) |
| **Plasmids used in the study** | | |
| **Use** | **Details** | **Reference** |
| PCR template for sgRNA | pICH86966::AtU6p::sgRNA_PDS | Nekrasov et al., 2013 |
| PCR template for pGlpT, sfGFP, and sfGFP-Ter | pYTK001 (Addgene #65108) | Lee et al., 2015 |
| Source of AtU6 promoter | pICSL01009::AtU6p | Nekrasov et al., 2013 |
| Source of PmCDA1-1xUGI | PmCDA1-1xUGI (Addgene #79620) | Nishida et al., 2016 |
| Source of evoCDA1 and 2xUGI | evoCDA1 pBT277 (Addgene #122608) | Thuronyi et al., 2019 |
| Source of APOBEC3A | A3A-PBE-ΔUGI (Addgene #119770) | Zong et al., 2018 |
| Source of nCas9 | Level 1 hCas9 module (Addgene #49771) | Nekrasov et al., 2013 |
| Source of nCas9-NG | pBT375 (Addgene #125616) | Thuronyi et al., 2019 |
| L1 module for ABE8e-based A-to-G editing | pGlpT-ABE8e-nCas9-TerL3S2P21 | Shelake et al., 2022 |
| L1 module for ABE9e-based A-to-G editing | pGlpT-ABE9e-nCas9-TerL3S2P21 | Shelake et al., 2022 |
| L1 module for Target-AID-based C-to-T editing | pGlpT-nCas9-PmCDA1-1xUGI-TerL3S2P21 | Shelake et al., 2022 |
| L1 module for evoCDA1-based C-to-T editing | pGlpT-evoCDA1-nCas9-2xUGI-TerL3S2P21 | Shelake et al., 2022 |
| L1 module for APOBEC3A-based C-to-T editing | pGlpT-A3A-nCas9-2xUGI-TerL3S2P21 | Shelake et al., 2022 |
| L1 module to design iACBE1 | pGlpT-ABE8e-nCas9- PmCDA1-1xUGI-TerL3S2P21 | This work |
| L1 module to design iACBE2 | pGlpT-evoCDA1-ABE8e-nCas9-2xUGI-TerL3S2P21 | This work |
| L1 module to design iACBE3 | pGlpT-APOBEC3A-ABE8e-nCas9-2xUGI-TerL3S2P21 | This work |
| L1 module to design iACBE4 | pGlpT-evoCDA1-ABE9e-nCas9-2xUGI-TerL3S2P21 | This work |
| L1 module to design iACBE2 (dCas9) | pGlpT-evoCDA1-ABE8e-dCas9-NG-2xUGI-TerL3S2P21 | This work |
| L1 module to test ABE8e-NG | pGlpT-ABE8e-(nCas9-NG)-TerL3S2P21 | This work |
| L1 module to test ABE9e-NG | pGlpT-ABE9e-(nCas9-NG)-TerL3S2P21 | This work |
| L1 module to test PmCDA1-NG | pGlpT-(nCas9-NG)-PmCDA1-1xUGI-TerL3S2P21 | This work |
| L1 module to test evoCDA1-NG | pGlpT-evoCDA1-(nCas9-NG)-2xUGI-TerL3S2P21 | This work |
| L1 module to test APOBEC3A-NG | pGlpT-A3A-(nCas9-NG)-2xUGI-TerL3S2P21 | This work |
| L1 module for iACBE4-NG | pGlpT-evoCDA1-ABE9e-(nCas9-NG)-2xUGI-TerL3S2P21 | This work |
| L1 module for iACBE2-NG | pGlpT-evoCDA1-ABE8e-(nCas9-NG)-2xUGI-TerL3S2P21 | This work |
| **Biopart sequences used for assembling dual base editing systems** | | |

| **pGlpT (BBa_J72163 GlpT+RBS) promoter**  GAAAGTGAAACGTGATTTCATGCGTCATTTTGAACATTTTGTAAATCTTATTTAATAATGTGTGCGGCAATTCACATTTAATTTATGAATGTTTTCTTAACATCGCGGCAACTCAAGAAACGGCAGGTTCGGATCTTAGCTACTAGAGAAAGAGGAGAAATACTAG |
| --- |
| **TerL3S2P21 terminator**  CTCGGTACCAAATTCCAGAAAAGAGGCCTCCCGAAAGGGGGGCCTTTTTTCGTTTTGGTCC |
| **>pAtU6**  TGATCAAAAGTCCCACATCGATCAGGTGATATATAGCAGCTTAGTTTATATAATGATAGAGTCGACATAGCG |
| **>nCas9 (D10A)**  ATGGATAAAAAGTATTCTATTGGTTTAGCCATCGGCACTAATTCCGTTGGATGGGCTGTCATAACCGATGAATACAAAGTACCTTCAAAGAAATTTAAGGTGTTGGGGAACACAGACCGTCATTCGATTAAAAAGAATCTTATCGGTGCCCTCCTATTCGATAGTGGCGAAACGGCAGAGGCGACTCGCCTGAAACGAACCGCTCGGAGAAGGTATACACGTCGCAAGAACCGAATATGTTACTTACAAGAAATTTTTAGCAATGAGATGGCCAAAGTTGACGATTCTTTCTTTCACCGTTTGGAAGAGTCCTTCCTTGTCGAAGAGGACAAGAAACATGAACGGCACCCCATCTTTGGAAACATAGTAGATGAGGTGGCATATCATGAAAAGTACCCAACGATTTATCACCTCAGAAAAAAGCTAGTTGACTCAACTGATAAAGCGGACCTGAGGTTAATCTACTTGGCTCTTGCCCATATGATAAAGTTCCGTGGGCACTTTCTCATTGAGGGTGATCTAAATCCGGACAACTCGGATGTCGACAAACTGTTCATCCAGTTAGTACAAACCTATAATCAGTTGTTTGAAGAGAACCCTATAAATGCAAGTGGCGTGGATGCGAAGGCTATTCTTAGCGCCCGCCTCTCTAAATCCCGACGGCTAGAAAACCTGATCGCACAATTACCCGGAGAGAAGAAAAATGGGTTGTTCGGTAACCTTATAGCGCTCTCACTAGGCCTGACACCAAATTTTAAGTCGAACTTCGACTTAGCTGAAGATGCCAAATTGCAGCTTAGTAAGGACACGTACGATGACGATCTCGACAATCTACTGGCACAAATTGGAGATCAGTATGCGGACTTATTTTTGGCTGCCAAAAACCTTAGCGATGCAATCCTCCTATCTGACATACTGAGAGTTAATACTGAGATTACCAAGGCGCCGTTATCCGCTTCAATGATCAAAAGGTACGATGAACATCACCAAGACTTGACACTTCTCAAGGCCCTAGTCCGTCAGCAACTGCCTGAGAAATATAAGGAAATATTCTTTGATCAGTCGAAAAACGGGTACGCAGGTTATATTGACGGCGGAGCGAGTCAAGAGGAATTCTACAAGTTTATCAAACCCATATTAGAGAAGATGGATGGGACGGAAGAGTTGCTTGTAAAACTCAATCGCGAAGATCTACTGCGAAAGCAGCGGACTTTCGACAACGGTAGCATTCCACATCAAATCCACTTAGGCGAATTGCATGCTATACTTAGAAGGCAGGAGGATTTTTATCCGTTCCTCAAAGACAATCGTGAAAAGATTGAGAAAATCCTAACCTTTCGCATACCTTACTATGTGGGACCCCTGGCCCGAGGGAACTCTCGGTTCGCATGGATGACAAGAAAGTCCGAAGAAACGATTACTCCATGGAATTTTGAGGAAGTTGTCGATAAAGGTGCGTCAGCTCAATCGTTCATCGAGAGGATGACCAACTTTGACAAGAATTTACCGAACGAAAAAGTATTGCCTAAGCACAGTTTACTTTACGAGTATTTCACAGTGTACAATGAACTCACGAAAGTTAAGTATGTCACTGAGGGCATGCGTAAACCCGCCTTTCTAAGCGGAGAACAGAAGAAAGCAATAGTAGATCTGTTATTCAAGACCAACCGCAAAGTGACAGTTAAGCAATTGAAAGAGGACTACTTTAAGAAAATTGAATGCTTCGATTCTGTCGAGATCTCCGGGGTAGAAGATCGATTTAATGCGTCACTTGGTACGTATCATGACCTCCTAAAGATAATTAAAGATAAGGACTTCCTGGATAACGAAGAGAATGAAGATATCTTAGAAGATATAGTGTTGACTCTTACCCTCTTTGAAGATCGGGAAATGATTGAGGAAAGACTAAAAACATACGCTCACCTGTTCGACGATAAGGTTATGAAACAGTTAAAGAGGCGTCGCTATACGGGCTGGGGACGATTGTCGCGGAAACTTATCAACGGGATAAGAGACAAGCAAAGTGGTAAAACTATTCTCGATTTTCTAAAGAGCGACGGCTTCGCCAATAGGAACTTTATGCAGCTGATCCATGATGACTCTTTAACCTTCAAAGAGGATATACAAAAGGCACAGGTTTCCGGACAAGGGGACTCATTGCACGAACATATTGCGAATCTTGCTGGTTCGCCAGCCATCAAAAAGGGCATACTCCAGACAGTCAAAGTAGTGGATGAGCTAGTTAAGGTCATGGGACGTCACAAACCGGAAAACATTGTAATCGAGATGGCACGCGAAAATCAAACGACTCAGAAGGGGCAAAAAAACAGTCGAGAGCGGATGAAGAGAATAGAAGAGGGTATTAAAGAACTGGGCAGCCAGATCTTAAAGGAGCATCCTGTGGAAAATACCCAATTGCAGAACGAGAAACTTTACCTCTATTACCTACAAAATGGAAGGGACATGTATGTTGATCAGGAACTGGACATAAACCGTTTATCTGATTACGACGTCGATCACATTGTACCCCAATCCTTTTTGAAGGACGATTCAATCGACAATAAAGTGCTTACACGCTCGGATAAGAACCGAGGGAAAAGTGACAATGTTCCAAGCGAGGAAGTCGTAAAGAAAATGAAGAACTATTGGCGGCAGCTCCTAAATGCGAAACTGATAACGCAAAGAAAGTTCGATAACTTAACTAAAGCTGAGAGGGGTGGCTTGTCTGAACTTGACAAGGCCGGATTTATTAAACGTCAGCTCGTGGAAACCCGCCAAATCACAAAGCATGTTGCACAGATACTAGATTCCCGAATGAATACGAAATACGACGAGAACGATAAGCTGATTCGGGAAGTCAAAGTAATCACTTTAAAGTCAAAATTGGTGTCGGACTTCAGAAAGGATTTTCAATTCTATAAAGTTAGGGAGATAAATAACTACCACCATGCGCACGACGCTTATCTTAATGCCGTCGTAGGGACCGCACTCATTAAGAAATACCCGAAGCTAGAAAGTGAGTTTGTGTATGGTGATTACAAAGTTTATGACGTCCGTAAGATGATCGCGAAAAGCGAACAGGAGATAGGCAAGGCTACAGCCAAATACTTCTTTTATTCTAACATTATGAATTTCTTTAAGACGGAAATCACTCTGGCAAACGGAGAGATACGCAAACGACCTTTAATTGAAACCAATGGGGAGACAGGTGAAATCGTATGGGATAAGGGCCGGGACTTCGCGACGGTGAGAAAAGTTTTGTCCATGCCCCAAGTCAACATAGTAAAGAAAACTGAGGTGCAGACCGGAGGGTTTTCAAAGGAATCGATTCTTCCAAAAAGGAATAGTGATAAGCTCATCGCTCGTAAAAAGGACTGGGACCCGAAAAAGTACGGTGGCTTCGATAGCCCTACAGTTGCCTATTCTGTCCTAGTAGTGGCAAAAGTTGAGAAGGGAAAATCCAAGAAACTGAAGTCAGTCAAAGAATTATTGGGGATAACGATTATGGAGCGCTCGTCTTTTGAAAAGAACCCCATCGACTTCCTTGAGGCGAAAGGTTACAAGGAAGTAAAAAAGGATCTCATAATTAAACTACCAAAGTATAGTCTGTTTGAGTTAGAAAATGGCCGAAAACGGATGTTGGCTAGCGCCGGAGAGCTTCAAAAGGGGAACGAACTCGCACTACCGTCTAAATACGTGAATTTCCTGTATTTAGCGTCCCATTACGAGAAGTTGAAAGGTTCACCTGAAGATAACGAACAGAAGCAACTTTTTGTTGAGCAGCACAAACATTATCTCGACGAAATCATAGAGCAAATTTCGGAATTCAGTAAGAGAGTCATCCTAGCTGATGCCAATCTGGACAAAGTATTAAGCGCATACAACAAGCACAGGGATAAACCCATACGTGAGCAGGCGGAAAATATTATCCATTTGTTTACTCTTACCAACCTCGGCGCTCCAGCCGCATTCAAGTATTTTGACACAACGATAGATCGCAAACGATACACTTCTACCAAGGAGGTGCTAGACGCGACACTGATTCACCAATCCATCACGGGATTATATGAAACTCGGATAGATTTGTCACAGCTTGGGGGTGAC |
| **>dCas9 (D10A+H140A)**  ATGGATAAAAAGTATTCTATTGGTTTAGCCATCGGCACTAATTCCGTTGGATGGGCTGTCATAACCGATGAATACAAAGTACCTTCAAAGAAATTTAAGGTGTTGGGGAACACAGACCGTCATTCGATTAAAAAGAATCTTATCGGTGCCCTCCTATTCGATAGTGGCGAAACGGCAGAGGCGACTCGCCTGAAACGAACCGCTCGGAGAAGGTATACACGTCGCAAGAACCGAATATGTTACTTACAAGAAATTTTTAGCAATGAGATGGCCAAAGTTGACGATTCTTTCTTTCACCGTTTGGAAGAGTCCTTCCTTGTCGAAGAGGACAAGAAACATGAACGGCACCCCATCTTTGGAAACATAGTAGATGAGGTGGCATATCATGAAAAGTACCCAACGATTTATCACCTCAGAAAAAAGCTAGTTGACTCAACTGATAAAGCGGACCTGAGGTTAATCTACTTGGCTCTTGCCCATATGATAAAGTTCCGTGGGCACTTTCTCATTGAGGGTGATCTAAATCCGGACAACTCGGATGTCGACAAACTGTTCATCCAGTTAGTACAAACCTATAATCAGTTGTTTGAAGAGAACCCTATAAATGCAAGTGGCGTGGATGCGAAGGCTATTCTTAGCGCCCGCCTCTCTAAATCCCGACGGCTAGAAAACCTGATCGCACAATTACCCGGAGAGAAGAAAAATGGGTTGTTCGGTAACCTTATAGCGCTCTCACTAGGCCTGACACCAAATTTTAAGTCGAACTTCGACTTAGCTGAAGATGCCAAATTGCAGCTTAGTAAGGACACGTACGATGACGATCTCGACAATCTACTGGCACAAATTGGAGATCAGTATGCGGACTTATTTTTGGCTGCCAAAAACCTTAGCGATGCAATCCTCCTATCTGACATACTGAGAGTTAATACTGAGATTACCAAGGCGCCGTTATCCGCTTCAATGATCAAAAGGTACGATGAACATCACCAAGACTTGACACTTCTCAAGGCCCTAGTCCGTCAGCAACTGCCTGAGAAATATAAGGAAATATTCTTTGATCAGTCGAAAAACGGGTACGCAGGTTATATTGACGGCGGAGCGAGTCAAGAGGAATTCTACAAGTTTATCAAACCCATATTAGAGAAGATGGATGGGACGGAAGAGTTGCTTGTAAAACTCAATCGCGAAGATCTACTGCGAAAGCAGCGGACTTTCGACAACGGTAGCATTCCACATCAAATCCACTTAGGCGAATTGCATGCTATACTTAGAAGGCAGGAGGATTTTTATCCGTTCCTCAAAGACAATCGTGAAAAGATTGAGAAAATCCTAACCTTTCGCATACCTTACTATGTGGGACCCCTGGCCCGAGGGAACTCTCGGTTCGCATGGATGACAAGAAAGTCCGAAGAAACGATTACTCCATGGAATTTTGAGGAAGTTGTCGATAAAGGTGCGTCAGCTCAATCGTTCATCGAGAGGATGACCAACTTTGACAAGAATTTACCGAACGAAAAAGTATTGCCTAAGCACAGTTTACTTTACGAGTATTTCACAGTGTACAATGAACTCACGAAAGTTAAGTATGTCACTGAGGGCATGCGTAAACCCGCCTTTCTAAGCGGAGAACAGAAGAAAGCAATAGTAGATCTGTTATTCAAGACCAACCGCAAAGTGACAGTTAAGCAATTGAAAGAGGACTACTTTAAGAAAATTGAATGCTTCGATTCTGTCGAGATCTCCGGGGTAGAAGATCGATTTAATGCGTCACTTGGTACGTATCATGACCTCCTAAAGATAATTAAAGATAAGGACTTCCTGGATAACGAAGAGAATGAAGATATCTTAGAAGATATAGTGTTGACTCTTACCCTCTTTGAAGATCGGGAAATGATTGAGGAAAGACTAAAAACATACGCTCACCTGTTCGACGATAAGGTTATGAAACAGTTAAAGAGGCGTCGCTATACGGGCTGGGGACGATTGTCGCGGAAACTTATCAACGGGATAAGAGACAAGCAAAGTGGTAAAACTATTCTCGATTTTCTAAAGAGCGACGGCTTCGCCAATAGGAACTTTATGCAGCTGATCCATGATGACTCTTTAACCTTCAAAGAGGATATACAAAAGGCACAGGTTTCCGGACAAGGGGACTCATTGCACGAACATATTGCGAATCTTGCTGGTTCGCCAGCCATCAAAAAGGGCATACTCCAGACAGTCAAAGTAGTGGATGAGCTAGTTAAGGTCATGGGACGTCACAAACCGGAAAACATTGTAATCGAGATGGCACGCGAAAATCAAACGACTCAGAAGGGGCAAAAAAACAGTCGAGAGCGGATGAAGAGAATAGAAGAGGGTATTAAAGAACTGGGCAGCCAGATCTTAAAGGAGCATCCTGTGGAAAATACCCAATTGCAGAACGAGAAACTTTACCTCTATTACCTACAAAATGGAAGGGACATGTATGTTGATCAGGAACTGGACATAAACCGTTTATCTGATTACGACGTCGATCACATTGTACCCCAATCCTTTTTGAAGGACGATTCAATCGACAATAAAGTGCTTACACGCTCGGATAAGAACCGAGGGAAAAGTGACAATGTTCCAAGCGAGGAAGTCGTAAAGAAAATGAAGAACTATTGGCGGCAGCTCCTAAATGCGAAACTGATAACGCAAAGAAAGTTCGATAACTTAACTAAAGCTGAGAGGGGTGGCTTGTCTGAACTTGACAAGGCCGGATTTATTAAACGTCAGCTCGTGGAAACCCGCCAAATCACAAAGCATGTTGCACAGATACTAGATTCCCGAATGAATACGAAATACGACGAGAACGATAAGCTGATTCGGGAAGTCAAAGTAATCACTTTAAAGTCAAAATTGGTGTCGGACTTCAGAAAGGATTTTCAATTCTATAAAGTTAGGGAGATAAATAACTACCACCATGCGCACGACGCTTATCTTAATGCCGTCGTAGGGACCGCACTCATTAAGAAATACCCGAAGCTAGAAAGTGAGTTTGTGTATGGTGATTACAAAGTTTATGACGTCCGTAAGATGATCGCGAAAAGCGAACAGGAGATAGGCAAGGCTACAGCCAAATACTTCTTTTATTCTAACATTATGAATTTCTTTAAGACGGAAATCACTCTGGCAAACGGAGAGATACGCAAACGACCTTTAATTGAAACCAATGGGGAGACAGGTGAAATCGTATGGGATAAGGGCCGGGACTTCGCGACGGTGAGAAAAGTTTTGTCCATGCCCCAAGTCAACATAGTAAAGAAAACTGAGGTGCAGACCGGAGGGTTTTCAAAGGAATCGATTCTTCCAAAAAGGAATAGTGATAAGCTCATCGCTCGTAAAAAGGACTGGGACCCGAAAAAGTACGGTGGCTTCGATAGCCCTACAGTTGCCTATTCTGTCCTAGTAGTGGCAAAAGTTGAGAAGGGAAAATCCAAGAAACTGAAGTCAGTCAAAGAATTATTGGGGATAACGATTATGGAGCGCTCGTCTTTTGAAAAGAACCCCATCGACTTCCTTGAGGCGAAAGGTTACAAGGAAGTAAAAAAGGATCTCATAATTAAACTACCAAAGTATAGTCTGTTTGAGTTAGAAAATGGCCGAAAACGGATGTTGGCTAGCGCCGGAGAGCTTCAAAAGGGGAACGAACTCGCACTACCGTCTAAATACGTGAATTTCCTGTATTTAGCGTCCCATTACGAGAAGTTGAAAGGTTCACCTGAAGATAACGAACAGAAGCAACTTTTTGTTGAGCAGCACAAACATTATCTCGACGAAATCATAGAGCAAATTTCGGAATTCAGTAAGAGAGTCATCCTAGCTGATGCCAATCTGGACAAAGTATTAAGCGCATACAACAAGCACAGGGATAAACCCATACGTGAGCAGGCGGAAAATATTATCCATTTGTTTACTCTTACCAACCTCGGCGCTCCAGCCGCATTCAAGTATTTTGACACAACGATAGATCGCAAACGATACACTTCTACCAAGGAGGTGCTAGACGCGACACTGATTCACCAATCCATCACGGGATTATATGAAACTCGGATAGATTTGTCACAGCTTGGGGGTGAC |
| **>ncas9-NG (D10A)**  ATGGACAAGAAGTACAGCATCGGCCTGGCCATCGGCACCAACTCTGTGGGCTGGGCCGTGATCACCGACGAGTACAAGGTGCCCAGCAAGAAATTCAAGGTGCTGGGCAACACCGACCGGCACAGCATCAAGAAGAACCTGATCGGAGCCCTGCTGTTCGACAGCGGCGAAACAGCCGAGGCCACCCGGCTGAAGAGAACCGCCAGAAGAAGATACACCAGACGGAAGAACCGGATCTGCTATCTGCAAGAGATCTTCAGCAACGAGATGGCCAAGGTGGACGACAGCTTCTTCCACAGACTGGAAGAGTCCTTCCTGGTGGAAGAGGATAAGAAGCACGAGCGGCACCCCATCTTCGGCAACATCGTGGACGAGGTGGCCTACCACGAGAAGTACCCCACCATCTACCACCTGAGAAAGAAACTGGTGGACAGCACCGACAAGGCCGACCTGCGGCTGATCTATCTGGCCCTGGCCCACATGATCAAGTTCCGGGGCCACTTCCTGATCGAGGGCGACCTGAACCCCGACAACAGCGACGTGGACAAGCTGTTCATCCAGCTGGTGCAGACCTACAACCAGCTGTTCGAGGAAAACCCCATCAACGCCAGCGGCGTGGACGCCAAGGCCATCCTGTCTGCCAGACTGAGCAAGAGCAGACGGCTGGAAAATCTGATCGCCCAGCTGCCCGGCGAGAAGAAGAATGGCCTGTTCGGAAACCTGATTGCCCTGAGCCTGGGCCTGACCCCCAACTTCAAGAGCAACTTCGACCTGGCCGAGGATGCCAAACTGCAGCTGAGCAAGGACACCTACGACGACGACCTGGACAACCTGCTGGCCCAGATCGGCGACCAGTACGCCGACCTGTTTCTGGCCGCCAAGAACCTGTCCGACGCCATCCTGCTGAGCGACATCCTGAGAGTGAACACCGAGATCACCAAGGCCCCCCTGAGCGCCTCTATGATCAAGAGATACGACGAGCACCACCAGGACCTGACCCTGCTGAAAGCTCTCGTGCGGCAGCAGCTGCCTGAGAAGTACAAAGAGATTTTCTTCGACCAGAGCAAGAACGGCTACGCCGGCTACATTGACGGCGGAGCCAGCCAGGAAGAGTTCTACAAGTTCATCAAGCCCATCCTGGAAAAGATGGACGGCACCGAGGAACTGCTCGTGAAGCTGAACAGAGAGGACCTGCTGCGGAAGCAGCGGACCTTCGACAACGGCAGCATCCCCCACCAGATCCACCTGGGAGAGCTGCACGCCATTCTGCGGCGGCAGGAAGATTTTTACCCATTCCTGAAGGACAACCGGGAAAAGATCGAGAAGATCCTGACCTTCCGCATCCCCTACTACGTGGGCCCTCTGGCCAGGGGAAACAGCAGATTCGCCTGGATGACCAGAAAGAGCGAGGAAACCATCACCCCCTGGAACTTCGAGGAAGTGGTGGACAAGGGCGCTTCCGCCCAGAGCTTCATCGAGCGGATGACCAACTTCGATAAGAACCTGCCCAACGAGAAGGTGCTGCCCAAGCACAGCCTGCTGTACGAGTACTTCACCGTGTATAACGAGCTGACCAAAGTGAAATACGTGACCGAGGGAATGAGAAAGCCCGCCTTCCTGAGCGGCGAGCAGAAAAAGGCCATCGTGGACCTGCTGTTCAAGACCAACCGGAAAGTGACCGTGAAGCAGCTGAAAGAGGACTACTTCAAGAAAATCGAGTGCTTCGACTCCGTGGAAATCTCCGGCGTGGAAGATCGGTTCAACGCCTCCCTGGGCACATACCACGATCTGCTGAAAATTATCAAGGACAAGGACTTCCTGGACAATGAGGAAAACGAGGACATTCTGGAAGATATCGTGCTGACCCTGACACTGTTTGAGGACAGAGAGATGATCGAGGAACGGCTGAAAACCTATGCCCACCTGTTCGACGACAAAGTGATGAAGCAGCTGAAGCGGCGGAGATACACCGGCTGGGGCAGGCTGAGCCGGAAGCTGATCAACGGCATCCGGGACAAGCAGTCCGGCAAGACAATCCTGGATTTCCTGAAGTCCGACGGCTTCGCCAACAGAAACTTCATGCAGCTGATCCACGACGACAGCCTGACCTTTAAAGAGGACATCCAGAAAGCCCAGGTGTCCGGCCAGGGCGATAGCCTGCACGAGCACATTGCCAATCTGGCCGGCAGCCCCGCCATTAAGAAGGGCATCCTGCAGACAGTGAAGGTGGTGGACGAGCTCGTGAAAGTGATGGGCCGGCACAAGCCCGAGAACATCGTGATCGAAATGGCCAGAGAGAACCAGACCACCCAGAAGGGACAGAAGAACAGCCGCGAGAGAATGAAGCGGATCGAAGAGGGCATCAAAGAGCTGGGCAGCCAGATCCTGAAAGAACACCCCGTGGAAAACACCCAGCTGCAGAACGAGAAGCTGTACCTGTACTACCTGCAGAATGGGCGGGATATGTACGTGGACCAGGAACTGGACATCAACCGGCTGTCCGACTACGATGTGGACCATATCGTGCCTCAGAGCTTTCTGAAGGACGACTCCATCGACAACAAGGTGCTGACCAGAAGCGACAAGAACCGGGGCAAGAGCGACAACGTGCCCTCCGAAGAGGTCGTGAAGAAGATGAAGAACTACTGGCGGCAGCTGCTGAACGCCAAGCTGATTACCCAGAGAAAGTTCGACAATCTGACCAAGGCCGAGAGAGGCGGCCTGAGCGAACTGGATAAGGCCGGCTTCATCAAGAGACAGCTGGTGGAAACCCGGCAGATCACAAAGCACGTGGCACAGATCCTGGACTCCCGGATGAACACTAAGTACGACGAGAATGACAAGCTGATCCGGGAAGTGAAAGTGATCACCCTGAAGTCCAAGCTGGTGTCCGATTTCCGGAAGGATTTCCAGTTTTACAAAGTGCGCGAGATCAACAACTACCACCACGCCCACGACGCCTACCTGAACGCCGTCGTGGGAACCGCCCTGATCAAAAAGTACCCTAAGCTGGAAAGCGAGTTCGTGTACGGCGACTACAAGGTGTACGACGTGCGGAAGATGATCGCCAAGAGCGAGCAGGAAATCGGCAAGGCTACCGCCAAGTACTTCTTCTACAGCAACATCATGAACTTTTTCAAGACCGAGATTACCCTGGCCAACGGCGAGATCCGGAAGCGGCCTCTGATCGAGACAAACGGCGAAACCGGGGAGATCGTGTGGGATAAGGGCCGGGATTTTGCCACCGTGCGGAAAGTGCTGAGCATGCCCCAAGTGAATATCGTGAAAAAGACCGAGGTGCAGACAGGCGGCTTCAGCAAAGAGTCTATCCGGCCCAAGAGGAACAGCGATAAGCTGATCGCCAGAAAGAAGGACTGGGACCCTAAGAAGTACGGCGGCTTCGTGAGCCCCACCGTGGCCTATTCTGTGCTGGTGGTGGCCAAAGTGGAAAAGGGCAAGTCCAAGAAACTGAAGAGTGTGAAAGAGCTGCTGGGGATCACCATCATGGAAAGAAGCAGCTTCGAGAAGAATCCCATCGACTTTCTGGAAGCCAAGGGCTACAAAGAAGTGAAAAAGGACCTGATCATCAAGCTGCCTAAGTACTCCCTGTTCGAGCTGGAAAACGGCCGGAAGAGAATGCTGGCCTCTGCCCGGTTCCTGCAGAAGGGAAACGAACTGGCCCTGCCCTCCAAATATGTGAACTTCCTGTACCTGGCCAGCCACTATGAGAAGCTGAAGGGCTCCCCCGAGGATAATGAGCAGAAACAGCTGTTTGTGGAACAGCACAAGCACTACCTGGACGAGATCATCGAGCAGATCAGCGAGTTCTCCAAGAGAGTGATCCTGGCCGACGCTAATCTGGACAAAGTGCTGTCCGCCTACAACAAGCACCGGGATAAGCCCATCAGAGAGCAGGCCGAGAATATCATCCACCTGTTTACCCTGACCAATCTGGGAGCCCCTCGGGCCTTCAAGTACTTTGACACCACCATCGACCGGAAGGTGTACCGGAGCACCAAAGAGGTGCTGGACGCCACCCTGATCCACCAGAGCATCACCGGCCTGTACGAGACACGGATCGACCTGTCTCAGCTGGGAGGTGAC |
| **>ABE8e-XTEN**  ATGAGTGAGGTGGAGTTCTCTCACGAATACTGGATGCGACATGCTCTAACGCTAGCAAAACGAGCGAGGGATGAACGAGAGGTTCCTGTAGGAGCAGTGTTGGTTCTGAACAACAGAGTTATTGGTGAAGGTTGGAATCGTGCTATTGGGCTTCACGACCCAACAGCCCATGCCGAAATAATGGCGCTCAGGCAAGGAGGCTTAGTAATGCAAAACTACAGATTAATCGACGCGACCCTGTATGTCACGTTCGAGCCATGCGTTATGTGCGCGGGCGCGATGATTCATTCTAGAATTGGAAGGGTTGTTTTTGGGGTGAGAAATTCTAAAAGAGGTGCGGCTGGGAGTCTTATGAATGTTCTCAATTACCCTGGTATGAACCATCGAGTGGAAATCACGGAGGGGATTTTGGCGGACGAATGTGCAGCATTGTTATGCGATTTCTATCGTATGCCTAGGCAGGTTTTCAACGCTCAGAAGAAGGCGCAAAGTTCAATTAATTCCGGCGGGTCCTCAGGAGGTAGCTCAGGGTCAGAGACTCCTGGAACATCTGAGTCAGCTACTCCTGAAAGCTCTGGAGGATCTTCTGGTGGTTCA |
| **>ABE9e-XTEN**  ATGAGTGAGGTGGAGTTCTCTCACGAATACTGGATGCGACATGCTCTAACGCTAGCAAAACGAGCGAGGGATGAACGAGAGGTTCCTGTAGGAGCAGTGTTGGTTCTGAACAACAGAGTTATTGGTGAAGGTTGGAATCGTGCTATTGGGCTTCACGACCCAACAGCCCATGCCGAAATAATGGCGCTCAGGCAAGGAGGCTTAGTAATGCAAAACTACAGATTAATCGACGCGACCCTGTATTCAACGTTCGAGCCATGCGTTATGTGCGCGGGCGCGATGATTCATTCTAGAATTGGAAGGGTTGTTTTTGGGGTGAGAAATTCTAAAAGAGGTGCGGCTGGGAGTCTTATGAATGTTCTCAATTACCCTGGTATGAACCATCGAGTGGAAATCACGGAGGGGATTTTGGCGGACGAATGTGCAGCATTGTTATGCGATTTCTATCGTATGCCTAGGAGAGTTTTCAACGCTCAGAAGAAGGCGCAAAGTTCAATTAATTCCGGCGGGTCCTCAGGAGGTAGCTCAGGGTCAGAGACTCCTGGAACATCTGAGTCAGCTACTCCTGAAAGCTCTGGAGGATCTTCTGGTGGTTCA |
| **>PmCDA1-1xUGI (SH3 Linker-3xFLAG-PmCDA1-1xUGI)**  GGTGGAGGAGGTACCGGCGGTGGAGGCTCAGCAGAATACGTACGAGCTCTGTTTGACTTCAATGGGAATGACGAGGAGGATCTCCCCTTTAAGAAGGGCGATATTCTCCGCATCAGAGATAAGCCCGAAGAACAATGGTGGAATGCCGAGGATAGCGAAGGGAAAAGGGGCATGATTCTGGTGCCATATGTGGAGAAATATTCCGGTGACTACAAAGACCATGATGGGGATTACAAAGACCACGACATCGACTACAAAGACGACGACGATAAATCAGGGATGACAGACGCCGAGTACGTGCGCATTCATGAGAAACTGGATATTTACACCTTCAAGAAGCAGTTCTTCAACAACAAGAAATCTGTGTCACACCGCTGCTACGTGCTGTTTGAGTTGAAGCGAAGGGGCGAAAGAAGGGCTTGCTTTTGGGGCTATGCCGTCAACAAGCCCCAAAGTGGCACCGAGAGAGGAATACACGCTGAGATATTCAGTATCCGAAAGGTGGAAGAGTATCTTCGGGATAATCCTGGGCAGTTTACGATCAACTGGTATTCCAGCTGGAGTCCTTGCGCTGATTGTGCCGAGAAAATTCTGGAATGGTATAATCAGGAACTTCGGGGAAACGGGCACACATTGAAAATCTGGGCCTGCAAGCTGTACTACGAGAAGAATGCCCGGAACCAGATAGGACTCTGGAATCTGAGGGACAATGGTGTAGGCCTGAACGTGATGGTTTCCGAGCACTATCAGTGTTGTCGGAAGATTTTCATCCAAAGCTCTCATAACCAGCTCAATGAAAACCGCTGGTTGGAGAAAACACTGAAACGTGCGGAGAAGTGGAGATCCGAGCTGAGCATCATGATCCAGGTCAAGATTCTGCATACCACTAAGTCTCCAGCCGTTGGTCCCAAGAAGAAAAGAAAAGTCGGTACCATGACCAACCTTTCCGACATCATAGAGAAGGAAACAGGCAAACAGTTGGTCATCCAAGAGTCGATACTCATGCTTCCTGAAGAAGTTGAGGAGGTCATTGGGAATAAGCCGGAAAGTGACATTCTCGTACACACTGCGTATGATGAGAGCACCGATGAGAACGTGATGCTGCTCACGTCAGATGCCCCAGAGTACAAACCCTGGGCTCTGGTGATTCAGGACTCTAATGGAGAGAACAAGATCAAGATGCTATAA |
| **>evoCDA1-XTEN**  AGTACCGACGCCGAGTACGTGCGGATCCACGAGAAGCTGGATATCTATACATTCAAGAAGCAGTTTAGCAACAATAAGAAGTCCGTGTCTCACAGATGCTACGTGCTGTTCGAGCTGAAGCGGAGAGGAGAGAGGCGCGCCTGTTTTTGGGGCTATGCCGTGAACAAGCCACAGTCTGGAACCGAGAGGGGAATCCACGCAGAGATCTTCAGCATCAGGAAGGTGGAGGAGTACCTGCGCGACAACCCCGGCCAGTTTACAATCAATTGGTATAGCTCCTGGAGCCCTTGCGCCGATTGTGCCGAGAAGATCCTGGAGTGGTACAACCAGGAGCTGAGGGGCAATGGCCACACCCTGAAGATCTGGGTGTGCAAGCTGTACTATGAGAAGAACGCCAGGAATCAGATCGGCCTGTGGAACCTGCGCGACAATGGCGTGGGCCTGAACGTGATGGTGTCCGAGCACTATCAGTGCTGTCGCAAGATCTTTATCCAGTCTAGCCACAATCAGCTGAACGAGAATCGGTGGCTGGAGAAAACACTGAAGAGAGCCGAGAAGCGGAGAAGCGAGCTGTCCATCATGTTTCAGGTGAAGATCCTGCACACCACAAAGTCTCCCGCCGTGTCTGGCGGATCTAGCGGAGGATCCTCTGGCAGCGAGACACCAGGAACAAGCGAGTCAGCAACACCAGAGAGCAGTGGCGGCAGCAGCGGCGGCAGC |
| **>Link-2xUGI**  TCTGGTGGTTCTGGTTCGAGCGGAGGATCCGGAGGATCTGGAGGCAGCGCTTCTAGCGGGGGGAGCACTAATCTGAGCGACATCATTGAGAAGGAGACTGGGAAACAGCTGGTCATTCAGGAGTCCATCCTGATGCTGCCTGAGGAGGTGGAGGAAGTGATCGGCAACAAGCCAGAGTCTGACATCCTGGTGCACACCGCCTACGACGAGTCCACAGATGAGAATGTGATGCTGCTGACCTCTGACGCCCCCGAGTATAAGCCTTGGGCCCTGGTCATCCAGGATTCTAACGGCGAGAATAAGATCAAGATGCTGAGCGGAGGATCCGGAGGATCTGGAGGCAGCACCAACCTGTCTGACATCATCGAGAAGGAGACAGGCAAGCAGCTGGTCATCCAGGAGAGCATCCTGATGCTGCCCGAAGAAGTCGAAGAAGTGATCGGAAACAAGCCTGAGAGCGATATCCTGGTCCATACCGCCTACGACGAGAGTACCGACGAAAATGTGATGCTGCTGACATCTGACGCCCCAGAGTATAAGCCCTGGGCTCTGGTCATCCAGGATTCCAACGGAGAGAACAAAATCAAAATGCTG |
| **>APOBEC3A-XTEN**  GAGGCCAGCCCGGCTAGCGGCCCAAGGCATCTCATGGACCCGCACATCTTCACCAGCAACTTCAACAACGGCATCGGCAGGCACAAGACCTACTTGTGCTACGAGGTGGAGAGGCTCGACAACGGAACCTCCGTGAAGATGGACCAACACAGGGGGTTCCTCCACAACCAAGCCAAGAACCTCCTCTGCGGCTTCTACGGCAGGCACGCCGAGTTGAGGTTCCTCGACTTGGTGCCATCCCTCCAACTCGATCCAGCCCAAATCTACCGCGTGACCTGGTTCATCTCCTGGTCCCCATGCTTCTCCTGGGGTTGCGCCGGCGAGGTTCGGGCTTTCCTCCAAGAAAACACCCACGTCCGCCTCCGCATTTTCGCCGCCAGGATCTATGATTACGACCCTCTCTACAAGGAGGCCCTCCAGATGCTGCGGGACGCCGGTGCTCAGGTGAGTATCATGACCTACGACGAGTTCAAGCACTGCTGGGACACCTTCGTTGACCACCAGGGCTGCCCATTCCAACCATGGGACGGTCTGGATGAACACAGCCAAGCCTTGTCCGGCAGGCTCCGGGCCATCCTCCAAAACCAGGGGAACTCCGGGAGCGAGACGCCAGGCACCTCCGAGTCGGCCACCCCAGAATCTCTTAAG |
